# Molecular dynamics-guided optimization of BGM0504 enhances dual-target agonism for combating diabetes and obesity

**DOI:** 10.1038/s41598-024-66998-8

**Published:** 2024-07-19

**Authors:** Jiandong Yuan, Wenlang Liu, Xiaohui Jiang, Yangqing Huang, Leilei Zong, Haifeng Ding, Xinyi Shen, Yujia Sun, Xiangyang Feng, Xionghao Li, Yunsong Song, Jianing Gu, Yuhuai Wang, Hao Liu, Zheng Zheng

**Affiliations:** 1 BrightGene Bio-Medical Technology Co., Ltd, Suzhou, 215000 People’s Republic of China; 2 Divamics Inc., Suzhou, 215000 People’s Republic of China; 3 The Sweetwood Group LLC, Rockville, MD 20850 USA; 4 School of Chemistry, Chemical Engineering and Life Science, Wuhan University of Technology, Wuhan, 430070 Hubei People’s Republic of China

**Keywords:** Molecular dynamics, GLP-1R/GIPR, Agonist peptide, Diabetes and obesity, NASH, Drug development, Computational chemistry, Structure-based drug design

## Abstract

The dual activation of glucagon-like peptide-1 receptor (GLP-1R) and glucose-dependent insulinotropic polypeptide receptor (GIPR) has emerged as a promising therapeutic strategy for managing type 2 diabetes and obesity. Tirzepatide, a dual agonist peptide, has exhibited superior clinical efficacy in glycemic and weight control compared to selective GLP-1R agonists. Nevertheless, the structural basis of Tirzepatide's extended half-life, attributed to an acylation side chain on the parent peptide, raises questions regarding its partial agonistic activity. Employing molecular dynamics simulations, we explored the dynamic processes of peptide-receptor interactions. We uncovered a crucial salt bridge between parent peptide and GLP-1R/GIPR at K20, a feature not discernible in cryo-electron microscopy structures. Building upon these insights, we developed an optimization strategy based on the parent peptide which involved repositioning the acylation side chain. The results of both in vitro and in vivo experiments demonstrated that the optimized peptide has twofold to threefold increase in agonistic activity compared to Tirzepatide while maintaining its extended half-life in plasma. This led to the design of BGM0504, which proved to be more effective than its predecessor, Tirzepatide, in both laboratory and animal studies.

## Introduction

The high prevalence of obesity and type 2 diabetes mellitus (T2DM) poses serious challenges to international health and socioeconomic progress^1–3^. Due to the interrelatedness of these two diseases, various treatments, including medication, insulin, and surgery, are widely used^4,5^. Furthermore, both diseases, diabetes and obesity, have been shown to be risk factors for a number of related diseases, including microbial and macrovascular complications and nonalcoholic fatty liver disease (NAFLD)^6^.

Glucagon-like peptide-1 (GLP-1)^7^ is a natural hormone produced by intestinal L cells after consuming food. It can help to avoid the risk of hypoglycemia, which can occur with insulin administration. GLP-1^8–11^ has shown potential in treating diabetes and obesity. GLP-1R^12^ agonists were initially developed to mimic GLP-1 and activate GLP-1R. However, higher doses of these agonists have been associated with gastrointestinal side effects, which can make it difficult for patients to achieve their glycemic control and weight management goals^13^. To overcome these complications, researchers have explored alternative treatment strategies. One promising strategy is to optimize the GLP-1 RA sequence to achieve a similar agonism effect against the glucose-dependent insulinotropic polypeptide receptor (GIPR). This can reduce the reliance on GLP-1-mediated effects and avoid gastrointestinal side effects^14,15^. The most promising drug with GLP-1R/GIPR dual agonist effects is Tripeptide (LY3298176), developed by Eli Lilly and Company, which the Food and Drug Administration (FDA) has approved for treating type II diabetes and obesity^16^.

As a dual agonist targeting GLP-1R/GIPR, Tirzepatide has demonstrated significant effectiveness in treating obesity and overweight^17^. In recent years, several research groups have employed structural biology techniques, especially cryo-electron microscopy (cryo-EM), to explore how Tirzepatide binds to and activates GLP-1R/GIPR. These studies have uncovered key residues and crucial interactions essential for its functionality. Scientists at Eli Lilly and Company introduced acylation at K20 with a γGlu-2 × OEG linker and C18 fatty diacid moiety, to increase the stability and prolong the half-life of Tirzepatide in human plasma. However, recent studies by Sun et al. and Zhao et al. revealed that the non-acylated Tirzepatide has higher potency in stimulating cAMP accumulation on GLP-1R/GIPR^18,19^. However, no research has been discovered yet to explore why the acylated moiety reduces the potency of the non-acylated Tirzepatide in stimulating cAMP accumulation at the molecular level.

In this study, we designed a novel GLP-1R/GIPR dual agonist named BGM0504 by an in-depth analysis of the interactions between the non-acylated Tirzepatide and GLP-1R/GIPR complexes using molecular dynamics simulations. Our analysis revealed favorable salt bridges between the K20 residue of the parent peptide and two glutamic acid residues on the EDC of both GLP-1R and GIPR. This finding underscores the crucial role of the K20 residue in mediating interactions during the binding of the non-acylated Tirzepatide to GLP-1R/GIPR, thereby augmenting the agonistic activity of the non-acylated Tirzepatide. Consequently, any acylation modification of the K20 residue is anticipated to disrupt such salt bridge formation, leading to a reduction in agonistic activity. In the design of BGM0504, we preserved the free amino group of the K20 residue while shifting the acylation site to the peptide C-terminal to mitigate the impact on the α-helical structure of the non-acylated Tirzepatide and its binding to GLP-1R/GIPR, thereby achieving balanced effects for both agonistic activity on the both receptors and prolonged half-life in the human plasma. Subsequently, Tirzepatide served as a positive control for the in vitro assessments of cAMP accumulation activity and binding affinity of BGM0504. Additionally, we conducted in vivo efficacy evaluations using mouse models of type II diabetes and Nonalcoholic Steatohepatitis (NASH) disease. Finally, comprehensive pharmacokinetic studies of BGM0504, including investigations in rats and cynomolgus monkeys, were undertaken.

## Results and discussion

### Structure analysis of Tirzepatide

As a tool for studying the conformational distributions of biomolecules, molecular dynamics simulation methods have shown reliable performance in studying multiple conformational states and conformational changing mechanisms for biomolecules^19–21^. In our molecular dynamics simulation by Amber18^22^, MOE2022^23^, Antechamber^24,25^, and membrane build by CHARMM-GUI^26^, the PDB files produced using CHARMM-GUI were converted to the Amber format using the charmmlipid2amber.py script integrated into Amber18, the AMBERff15ipq-m^27^, lipid14^28^, and the general Amber force field 2 (GAFF2)^29^ were applied for the protein, POPC membrane, and ligand molecule, AM1-BCC^25^, SHAKE^30^, PME^31,32^, CPPTRAJ^33^ algorithm or method was used. (See the “Supplementary Information” (SI) for details.)

**Figure 1:**
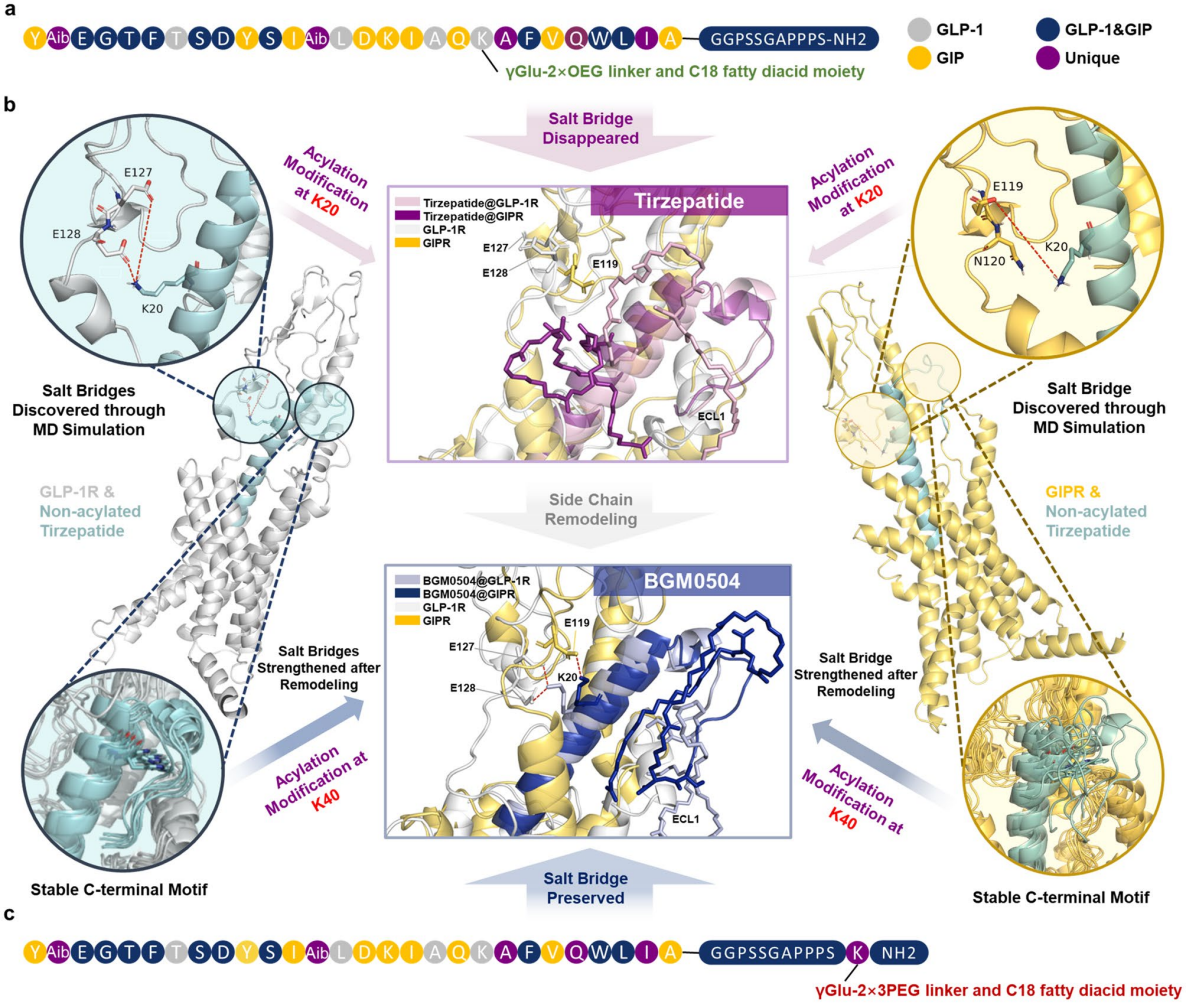
Structure Analysis and Optimization Strategy of Tirzepatide (**a**) Tirzepatide sequence and its side chain modification position (γGlu-2 × OEG linker and C18 fatty diacid moiety); different colors indicate sources and different natural peptides. (**b**) Comparison of acylation modification between Tirzepatide and BGM0504: On the left/right, the binding structure between the parent peptide and GLP-1/GIP, as well as the enlarged structure at K20 and the flexible stable region; in the middle, BGM0504 moves the modified side chain from K20 of Tirzepatide to K40. (**c**) BGM0504 sequence and its side chain modification (γGlu-2 × 3PEG linker and C18 fatty diacid moiety) position at the flexible stable region.

To elucidate the reasons for the reduced potency at stimulating cAMP accumulation of the non-acylated Tirzepatide after K20 acylation modification, we analyzed the structures of GLP-1R/non-acylated Tirzepatide (PDB: 7VBI) and GIPR/non-acylated Tirzepatide (PDB: 7FIY) complex^20^. The result revealed that K20 of the non-acylated Tirzepatide can form a salt bridge with the E128ECD of GLP-1R, but no significant beneficial interaction is seen between K20 non-acylated Tirzepatide and GIPR, as shown in Fig. 1. From a structure–activity relationship perspective, acylation modification at K20 leads to the loss of the salt bridge of E128ECD-K20 non-acylated Tirzepatide. We speculate that explains the non-acylated Tirzepatide has higher potency in stimulating cAMP accumulation on GIPR. However, in the GIPR/non-acylated Tirzepatide complex, K20 does not exhibit a direct, significant interaction with GIPR, thereby does not explaining the non-acylated Tirzepatide has higher potency in stimulating cAMP accumulation on GIPR.

Based on this hypothesis, we utilized the two sets of complexes mentioned as initial conformations and conducted separate 500 ns molecular dynamics simulations (excluding G protein and Nb35, which peptide-receptor complexes prepared with MOE2022) with three parallel runs. The root mean square deviation (RMSD), reflecting the average distance between CA atoms of superimposed protein/peptide structures and indicating the degree of similarity between two structures, demonstrated that the non-acylated Tirzepatide maintained stable binding at the GLP-1R/GIPR binding sites. However, it is also observed that the stability of the GIPR complex was not as robust as that of the GLP-1R complex. This observation arises from conformational fluctuations in the ECD region, and the peptide induced by the flexible structure of ECL1 in GIPR. In the following paragraphs, all binding free energy values and salt bridge occupancy are averaged over three parallel MD simulations.

Furthermore, as shown in Fig. 1b (upper left), we found that in GLP-1R, E128ECD-K20 non-acylated Tirzepatide and E127ECD-K20 non-acylated Tirzepatide form salt bridge. The occupancy of these salt bridges, representing the percentage of frames in which it occurs, is recorded as 88.3% and 80.7%. Interestingly, in the molecular dynamics simulation trajectory analysis of the GIPR complex, we observed that the occurrence of the salt bridge in E119ECD-K20 non-acylated Tirzepatide is recorded as 66.5%. K20 non-acylated Tirzepatide in the GLP-1R complex can form a salt bridge with both E128ECD and E127ECD, while in the GIPR complex, K20 non-acylated Tirzepatide can form a salt bridge with E119ECD, as shown in Fig. 1b (upper right). The computed binding free energies using MMGBSA showed that non-acylated Tirzepatide exhibited superior binding affinity compared to Tirzepatide for both GLP-1R (− 179.06 vs. − 161.38 kcal/mol) and GIPR (− 160.59 vs. − 152.82 kcal/mol) (See Supplementary Table 1).This result provided a molecular dynamic-based explanation for why the acylation modification of Tirzepatide’s K20 resulted in the reduction of corresponding GLP-1R/GIPR potency of the non-acylated Tirzepatide at stimulating cAMP accumulation (See Supplementary Table 2).

### Optimization of Tirzepatide and in vitro experimental evaluation

In light of our research and existing literature, the acylation modification of K20 did not fully exploit the agonistic activity potential of the Tirzepatide parent peptide. To address this limitation, we first conducted a preliminary analysis to assess the suitability of K16 in Tirzepatide as a candidate residue for acylation modification. The structural sequence of Tirzepatide (Fig. 1a) reveals that the K16 residue is derived from the natural GIP sequence. Past studies often utilized K16 to enhance the agonistic activity of dual agonist peptides targeting GIPR^14,34,35^. Consequently, acylation modification at K16 may diminish the agonistic effect on GIPR. Additionally, a structure–activity relationship analysis^33^ demonstrated that K16 can form a cation-pi interaction with F127 of GIPR, with the occurrence of this interaction recorded at 61.0% in molecular dynamics simulations. Therefore, introducing acylation modification at the K16 site might impact this essential interaction, reducing GIPR agonistic activity for the peptide.

Then, building upon these findings, introducing acylated side chains (γGlu-2 × OEG linker and C18 fatty diacid moiety) becomes imperative to enhance half-life in plasma. However, under this premise, acylation modification of the existing lysine residues in the Tirzepatide parent peptide may reduce agonistic activities on GLP-1R/GIPR. Consequently, we explored the feasibility of appending a lysine (K) to the C-terminal of the Tirzepatide parent peptide to introduce acylated moiety as shown in Fig. 1b (middle). Tirzepatide is recognized to possess nine additional residues at the C-terminal, derived from the exendin-4 sequence. Previous studies revealed that, compared to GLP-1, exendin-4 features nine extra residues at the C-terminal, forming a Trp cage motif that establishes a stable folding structure, shielding Trp from external influences^36^.

As shown in Fig. 1b (lower right), in comparison to GIPR, the non-acylated Tirzepatide exhibits a more stable set of C-terminal conformations on GLP-1R, possibly influenced by the distinct flexibility of ECL1 in GLP-1R/GIPR. However, from an overall stability perspective, the C-terminal can maintain a relatively stable folded structure after binding to GLP-1R/GIPR. Therefore, as shown in Fig. 1c, we posit that adding a K to the C-terminal of the Tirzepatide parent peptide to introduce acylated moiety is a feasible strategy.

**Figure 2:**
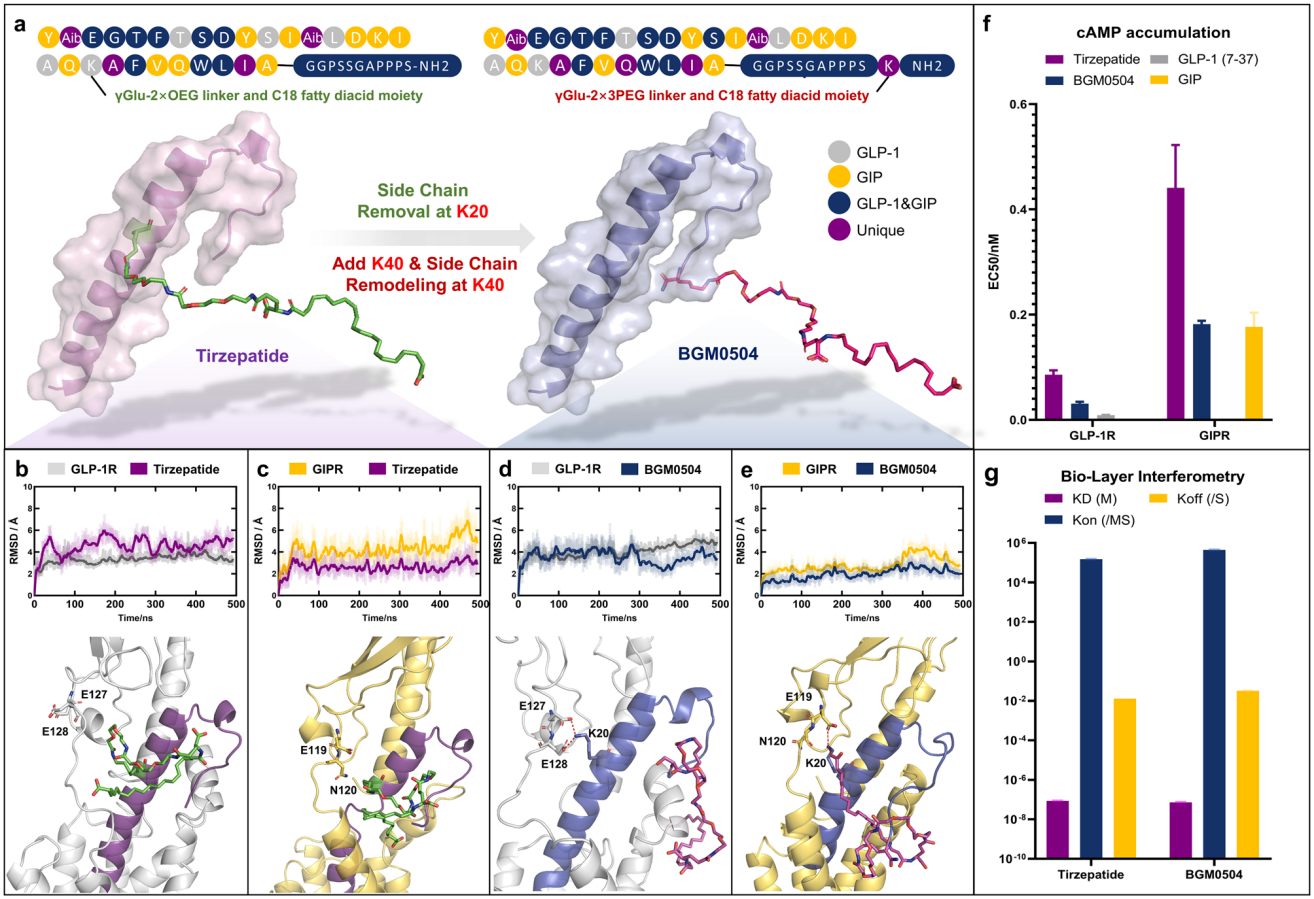
Structure Analysis and Performance Testing of BGM0504. (**a**) BGM0504 adds K40 amino acids on the basis of Tirzepatide and moves the side chain originally located at K20 to K40. (**b**) RMSD protein and peptide CA plot in the GLP-1R/Tirzepatide complex simulation and enlarged 500 ns main structure. (**c**) RMSD protein and peptide CA plot in the GIPR/ Tirzepatide complex simulation and enlarged 500 ns main structure. (**d**) RMSD protein and peptide CA plot in the GLP-1R/BGM0504 complex simulation and enlarged 500 ns main structure. (**e**) RMSD protein and peptide CA plot in the GIPR/BGM0504 complex simulation and enlarged 500 ns main structure. (**f**) The EC50 result of cAMP accumulation test for BGM0504, Tirzepatide, GLP-1(7-37) and GIP. (**g**) Bio-Layer Interferometry (BLI) test for BGM0504 and Tirzepatide binding to Human Serum Albumin (HSA) protein.

It is noteworthy that prior research revealed the enhanced binding affinity of Tirzepatide to GLP-1R (Fig. 2b) facilitated by the acylated moiety, suggesting a potential enhancement of agonistic activity on GLP-1R^20^. However, upon analyzing the structures of GLP-1R/non-acylated Tirzepatide and GIPR/non-acylated Tirzepatide complexes (Fig. 1b), we observed that in contrast to the acylation modification at K20, modifying the C-terminal avoids the salt bridge between K20 and the receptor. Nonetheless, this C-terminal modification increases the distance from the receptor’s ECD region.

Based on these insights, we relocated the acylated moiety to the C-terminal, concurrently extending the length of the acylated moiety linker (substituting the original γGlu-2 × OEG with γGlu-2 × 3PEG, as seen in Fig. 2a) in BGM0504. We constructed complexes of Tirzepatide and BGM0504 with GLP-1R/GIPR, respectively, and conducted molecular dynamics simulations (as shown in Fig. 2b–e). In GLP-1R, the salt bridges E128ECD-K20 BGM0504 and E127ECD-K20 BGM0504 were found to form with occurrences of 93.1% and 92.5%, respectively. In GIPR, the salt bridge E119ECD-K20 BGM0504 formed with a 69.1% occurrence. The conformation of GLP-1R/BGM0504 and GIPR/BGM0504 reveals that the acylated side chains facilitate their interaction by respectively binding to the ECL1 of GLP-1R and the ECD of GIPR. MMGBSA calculations revealed that BGM0504 exhibits stronger binding affinity than Tirzepatide for both GLP-1R (− 182.39 vs. − 161.38 kcal/mol) and GIPR (− 166.96 vs. − 152.82 kcal/mol). Moreover, after relocating the acylated moiety, BGM0504 also showed competitive binding affinity compared to the non-acylated Tirzepatide for both GLP-1R (− 182.39 vs. − 179.06 kcal/mol) and GIPR (− 166.96 vs. − 160.59 kcal/mol) (See Supplementary Table 1). The results underscored the necessity of introducing acylated side chains to not only enhance half-life in plasma, and for BGM0504 to keep its agonistic activities on GLP-1R/GIPR. In subsequent investigations, we assessed the in vitro and in vivo agonistic activity and efficacy of BGM0504, complemented by pharmacokinetic studies.

### The context of in vitro assessments

We first scrutinized the cAMP accumulation effect of BGM0504, exploring its interaction with GLP1-1R/GIPR. The receptor membrane proteins, extracted from cell lines highly expressing GLP1-1R/GIPR, facilitated a comprehensive analysis. In addition, Bio-Layer Interferometry (BLI)^37^ analysis was used to investigate the binding affinity of Tirzepatide and BGM0504 with HSA protein.

In the cAMP accumulation experiment, as shown in Fig. 2f, BGM0504 exhibited robust agonistic effects on both GLP1-1R/GIPR. Across three independent, replicated experiments, the average EC50 values were 0.031 and 0.182 nM. Remarkably, the activity of BGM0504 on GLP1-1R/GIPR surpassed that of Tirzepatide by 2–3 times (EC50 values of 0.086 and 0.441 nM, respectively). The heightened activity observed in BGM0504 serves as compelling evidence for the precision of our rational design strategy, while also highlighting the crucial role of molecular dynamics simulations in the development of dual agonists targeting GLP-1R/GIPR.

Tirzepatide was engineered with the incorporation of fatty acid side chains to enhance its binding affinity to HSA protein in an effort to extend the half-life in human plasma. The analysis revealed significant differences in the binding modes of BGM0504 and Tirzepatide to HSA protein; however, the computed binding free energies indicated comparable affinities between the two (SI, Fig. 2).

BLI assays (Fig. 2g) corroborated these findings, demonstrating that BGM0504 and Tirzepatide bind to HSA protein with dissociation constants KD of 73.04 nM and 86.30 nM, respectively, suggesting similar binding affinities, in alignment with the molecular dynamics results. These in vitro findings underscore the dual enhancement of BGM0504’s agonistic activity on GLP-1R/GIPR, setting the stage for subsequent in vivo investigations, employing Tirzepatide as a positive control to further elucidate the compound’s efficacy in mouse disease models associated with type II diabetes and NASH.

### In vivo evaluation: experimental assessment in db/db Mice and STZ + HFD (high-fat)-induced C57 BL/6 Mice

#### Experimental assessment in db/db Mice

In the in vivo experimental evaluation involving db/db mice, the positive control group received a subcutaneous injection of Tirzepatide at a dose of 0.15 mg/kg. In contrast, the db/db-ND group received only the drug vehicle via subcutaneous injection. Three parallel experimental groups were established to explore the dose-dependent characteristics of BGM0504 in db/db mice: a low-dose group (0.05 mg/kg), a standard dose group (0.15 mg/kg), and a high-dose group (0.5 mg/kg), each comprising 11 db/db mice. Subsequent administrations were conducted every 3 days following the initial administration, and the experiment spanned 31 days.

**Figure 3:**
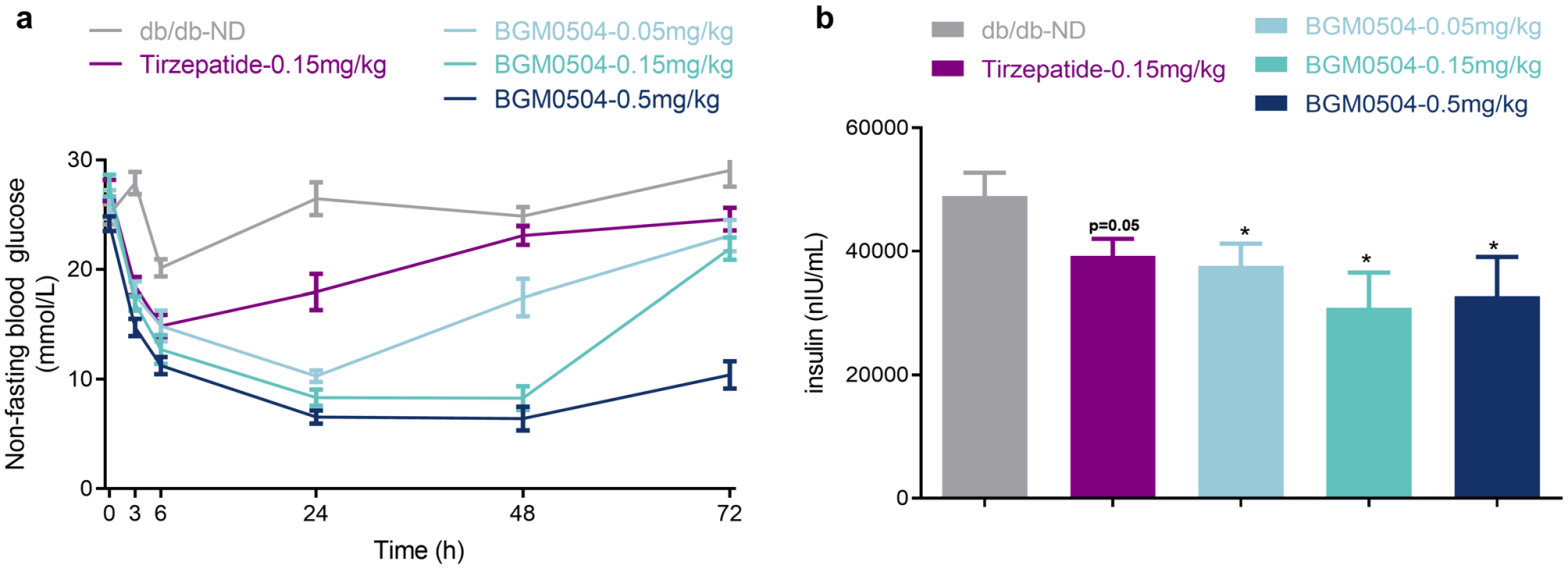
Experimental Assessment in db/db Mice. (**a**) The non-fasting blood glucose monitoring. (**b**) The mice serum insulin tests. (See Supplementary Table 3 for details.)

Within 72 h, the non-fasting blood glucose monitoring results (Fig. 3a) demonstrated significant blood glucose suppression in the Tirzepatide-0.15 mg/kg group and all BGM0504 dosing groups, compared to the db/db-ND group. After the initial administration, the Tirzepatide-0.15 mg/kg group experienced a rapid decrease in blood glucose within 6 h, followed by gradual increase over the next 66 h. Similarly, all three BGM0504 experimental groups exhibited rapid decrease within 24 h after the first administration. The low-dose and medium-dose groups showed gradual recovery and growth in the subsequent 48 h, while the high-dose group maintained relatively lower levels until 48 h and then displayed slow and small growth in the following 24 h. Notably, BGM0504’s blood glucose suppression effect demonstrated dose-dependency and superiority over the Tirzepatide-0.15 mg/kg group.

The results of the mice serum insulin tests (Fig. 3b) indicated a decrease in serum insulin concentrations in the Tirzepatide-0.15 mg/kg group and each of the BGM0504 dosing group. The decrease in serum insulin in each of the BGM0504 dosing group was statistically more significant compared to the control group (*p* ≤ 0.05). Furthermore, the decrease in serum insulin concentration in the BGM0504-0.15 mg/kg dosing group was significantly more effective than in the Tirzepatide-0.15 mg/kg dosing group.

Db/db mice, a model for type II diabetes^38^, had elevated blood glucose levels before administration. Tirzepatide effectively reduced blood glucose and insulin levels, validating the model. In our experiments, BGM0504 significantly lowered non-fasting blood glucose after the initial administration, surpassing Tirzepatide’s efficacy at an equivalent dose. Additionally, BGM0504 markedly diminished peripheral blood insulin. Notably, BGM0504 induced an acute decline in body weight and food intake during the early stages of administration, maintaining relative stability in later stages (Supplementary Fig. 5 and Fig. 6).

These results suggest that BGM0504 holds potential therapeutic promise, excelling in blood glucose control and impacting physiological parameters such as body weight and food intake in db/db mice. Further research endeavors will contribute to a more comprehensive understanding of BGM0504’s action mechanism and its potential applications in treating type II diabetes and obesity.

#### Experimental STZ + HFD-Induced C57 BL/6 mice

In our study, we evaluated the effects of positive drugs Tirzepatide and BGM0504 on diabetes and NASH models induced by STZ + HFD in C57 BL/6 mice. Both the control and model groups received a subcutaneous injection of the drug vehicle, while only the model group was fed a high-fat diet (HFD). The experimental results demonstrated similarities in reducing body weight and peripheral blood insulin, consistent with previous findings in db/db mice (see SI for detailed results).

**Figure 4:**
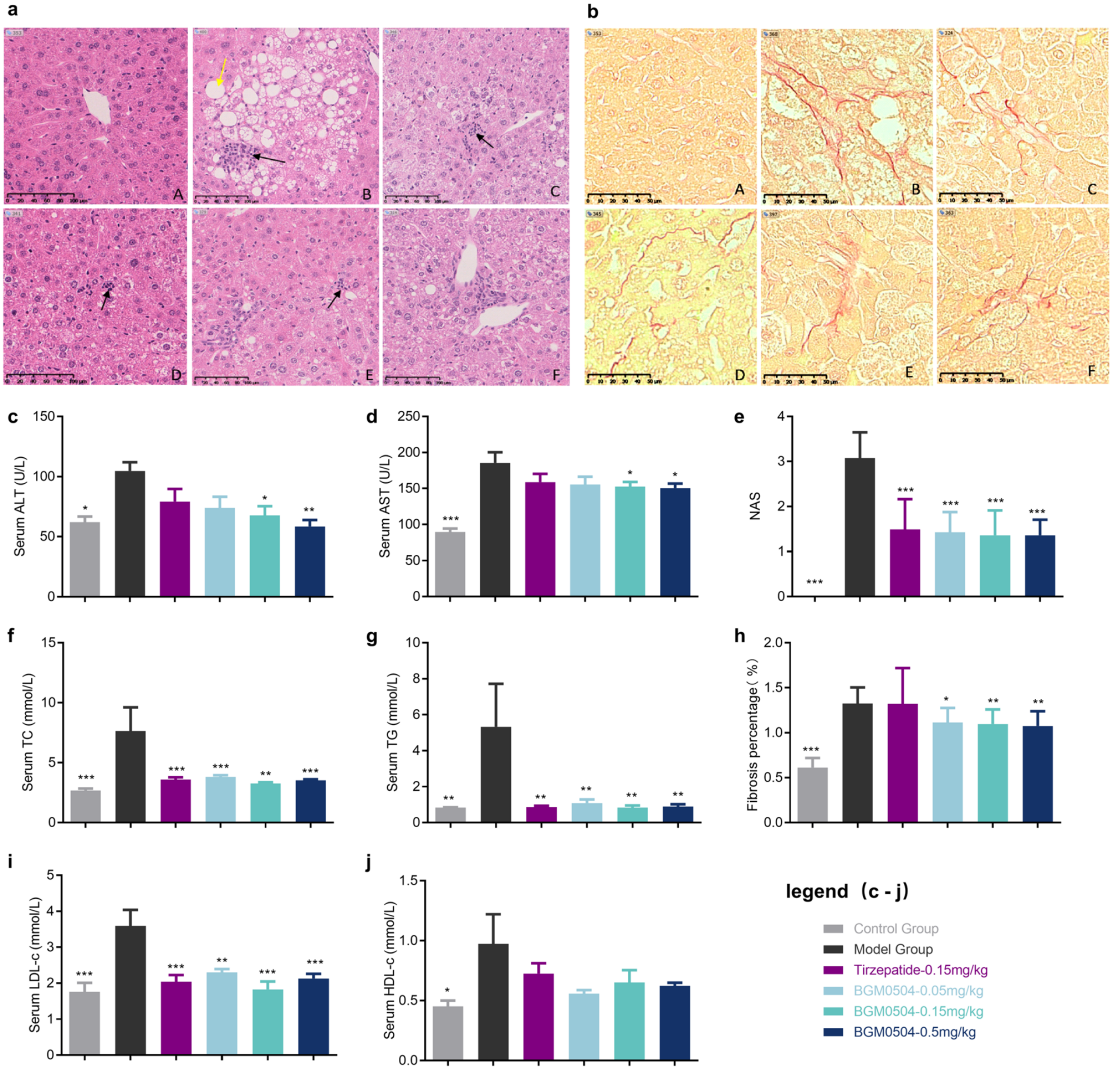
Experimental Assessment in STZ + HFD-Induced C57BL/6 Mice. (**a**) Hematoxylin & Eosin (H&E) staining in reducing liver adipose tissue damage for diabetes models. (**b**) Sirius red (SR) staining on liver tissue fibrosis for NASH models. (**c**) Alanine transaminase (ALT) level. (**d**) Aspartate aminotransferase (AST) level. (**e**) NAFLD Activity Score (NAS). (**f**) Liver total cholesterol (TC) level. (**g**) Liver triglyceride (TG) level. (**h**) Fibrosis rates level. (**i**) Low-density lipoprotein (LDL) level. (**j**) High-density lipoprotein (HDL) level. (***p < 0.001; **p < 0.01; *p < 0.05. See Supplementary Table 4–6 for details.)

For further exploration, we investigated the impact of Tirzepatide and BGM0504 on liver function and blood lipid levels. Serum levels of two liver injury markers, alanine transaminase (ALT) and aspartate aminotransferase (AST), were measured in the control group, model group, and four treated groups. Notably, all drug-dosing groups showed significant reductions in ALT and AST levels. BGM0504 exhibited dose-dependent effects, surpassing Tirzepatide in lowering ALT levels (Fig. 4c), while such trends were not quite significant for AST (Fig. 4d). In comparison to the model group, treated mice exhibited a marked reduction in liver TC (Fig. 4f) and triglyceride (TG) (Fig. 4g) levels. Remarkably, the TC and TG levels in the BGM0504-0.15 mg/kg treated mice had been decreased to those of control group mice. Notably, adipose tissue reduction was observed in mice treated with Tirzepatide-0.15 mg/kg and each dose of BGM0504, accompanied by a more significant decrease in LDL (Fig. 4i) levels than in HDL (Fig. 4j) levels. The BGM0504-0.15 mg/kg group exhibited an effect comparable to the average control level.

Our research findings underscore the potential of both Tirzepatide and BGM0504 in ameliorating dyslipidemia and attenuating hepatic damage to a certain degree. Significantly, when administered at equivalent doses, BGM0504 exhibited a notably superior therapeutic efficacy to Tirzepatide, emphasizing the advantageous potential of BGM0504 as a dual-target agonist of GLP-1R/GIPR. These discoveries provide robust experimental support for further exploration of the metabolic regulatory roles of BGM0504 and Tirzepatide.

H&E staining on liver tissue was conducted to investigate the potential of Tirzepatide and BGM0504 in reducing liver adipose tissue damage (Fig. 4a). In the model group, hepatocyte steatosis was evident as vacuolar degeneration of hepatocytes, which was extensively distributed throughout the liver lobes. Interlobular inflammation was characterized by scattered focal inflammatory cell infiltration within the liver lobules, with no hepatocyte ballooning observed (Fig. 4a and Supplementary Table 5). According to the NAS standards outlined in Supplementary Table 5, hepatocellular steatosis, interlobular inflammation, and NAS in the model group were markedly higher than those in the normal group (*p* < 0.001). Unlike humans, hepatocyte ballooning which signifies hepatocyte degeneration and cell death^39^, is exceptionally infrequent in rodent NASH models. Consequently, the NAFLD Activity Score (NAS) (Fig. 4e) primarily relies on assessing steatosis and inflammation^40^. In comparison to the model group, scores for hepatitis cell infiltration and hepatocyte steatosis were significantly reduced in the Tirzepatide-0.15 mg/kg group and each dose of the BGM0504 groups. Moreover, the total NAS in the Tirzepatide-0.15 mg/kg and BGM0504 groups at each dose was significantly lower than that in the model group (*p* < 0.001).

Hepatic fibrosis is a hallmark of NASH pathogenesis^41^, and NASH mice consistently displayed stable fibrosis. In this experiment, we employed SR staining to assess the impact of Tirzepatide and BGM0504 on liver tissue fibrosis (Fig. 4b). In the livers of mice in the model group, prominent SR staining indicated fibrosis primarily distributed in the portal vein, central vein, and intercellular areas. The semi-quantitative analysis of liver fibrosis (Supplementary Table 6) indicated that the fibrosis rate in the model group was 1.32 ± 0.02%, significantly higher than that in the normal control group (*p* < 0.001). In comparison to the model group, the fibrosis rates (Fig. 4h) in each dosage group of BGM0504 were significantly decreased. Notably, the fibrosis level in the mice of the BGM0504-0.15 mg/kg group reached 55% of the average level.

Our findings suggest the remarkable therapeutic efficacy of BGM0504 in the C57BL/6 mice who have diabetes with NASH induced by STZ + HFD. Across all dosage groups, it consistently reduces insulin, showing a clear dose-dependent response. Additionally, BGM0504 protects effect on the mice in the model group. Beyond glycemic control, it influences liver function and blood lipids, diminishing liver NAS and fibrosis rates while slowing NASH progression. Overall, BGM0504 effectively addresses diabetes combined with NASH, outperforming Tirzepatide at equivalent doses. These findings provide valuable insights for clinical applications and further research into BGM0504’s mechanisms of action.

#### Pharmacokinetic evaluation of BGM0504

The pharmacokinetic profile of the dual-target agonist BGM0504 was meticulously assessed through intravenous and subcutaneous administration in Sprague–Dawley (SD) rats and cynomolgus monkey models. A detailed exposition of the pharmacokinetic parameters is presented in the following table (Table 1). Upon intravenous injection of BGM0504-0.2 mg/kg in male and female SD rats, the plasma clearance rate was determined to be 0.0680 mL/min/kg, the volume of distribution at steady-state (Vd_ss_) was 0.0648 L/kg, and the elimination half-life (T_1/2_) was calculated as 13.6 h. These results suggest a predominant distribution in plasma with rapid absorption. After intravenous injection of 0.1 mg/kg BGM0504 in male and female cynomolgus monkeys, the plasma clearance rate was 0.0207 mL/min/kg, Vd_ss_ was 0.0608 L/kg, and T_1/2_ was 37.9 h.

**Table 1:** Pharmacokinetic profiles of BGM0504 in SD rats and cynomolgus monkeys after intravenous (i.v.) or subcutaneous (s.c.) injections.

	SD rats				Cynomolgus monkey			
Dose (mg/kg)	0.2 (i.v.) n = 6	0.3 (s.c.) n = 6	1.5 (s.c.) n = 6	7.5 (s.c.) n = 6	0.1 (i.v.) n = 6	0.2 (s.c.) n = 6	1.0 (s.c.) n = 6	5.0 (s.c.) n = 6
C_MAX_ (ng/mL)	4650 ± 411	814 ± 111	3830 ± 705	15,100 ± 1750	2860 ± 261	1440 ± 109	5970 ± 1090	33,500 ± 3060
T_MAX_ (h)	–	24.0 ± 0.0	24.0 ± 0.0	20.0 ± 6.2	–	16.0 ± 6.2	22.0 ± 14.0	20.0 ± 6.2
T_1/2_ (h)	13.6 ± 2.3	14.5 ± 1.4	13.4 ± 1.5	14.7 ± 2.4	37.9 ± 2.5	38.7 ± 1.8	41.7 ± 2.9	43.4 ± 5.9
Vd_ss_ (L/kg)	0.0648 ± 0.0058	–	–	–	0.0608 ± 0.0050	–	–	–
Cl (mL/min/kg)	0.0680 ± 0.0103	–	–	–	0.0207 ± 0.0022	–	–	–
AUC_0–last_ (ng h/mL)	38,800 ± 5150	14,100 ± 2440	63,900 ± 11,400	269,000 ± 43,000	34,600 ± 2470	26,100 ± 1740	109,000 ± 21,900	603,000 ± 94,200
AUC_0–∞_ (ng h/mL)	49,800 ± 7470	33,200 ± 6190	153,000 ± 27,700	637,000 ± 88,700	80,400 ± 7880	113,000 ± 7940	488,000 ± 51,200	2,720,000 ± 246,000
Bioavailability	–	44.5%	41.1%	34.3%	–	69.7%	60.5%	67.6%

–: not available.

Compared with the intravenous administration group, the bioavailability of 0.3, 1.5, and 7.5 mg/kg of BGM0504 in the male and female SD rats administered via a single subcutaneous injection was 44.5%, 41.1%, and 34.3%, respectively. The corresponding AUC_0-last_ values were 33,200, 153,000, and 637,000 ng·h/mL. In both male and female cynomolgus monkeys, following a single subcutaneous injection of 0.2, 1, and 5 mg/kg of BGM0504, the T_max_ ranged from 16 to 22 h, with elimination half-lives of 38.7, 41.7, and 43.4 h, respectively. In comparison with the intravenous administration group, the bioavailability of BGM0504 at 0.2, 1, and 5 mg/kg in male and female cynomolgus monkeys administered via a single subcutaneous injection was 69.7%, 60.5%, and 67.6%, respectively. The corresponding AUC_0-last_ values were 113,000, 488,000, and 2,720,000 ng·h/mL.

Overall, across the SD rat and cynomolgus monkey models, the Cmax ratio and AUC_0-last_ ratio among each dosing group closely approximated the dose ratio, with systemic exposure increasing proportionally to the administered dose. No discernible effects or gender differences were observed in any dosing group. These outcomes offer valuable pharmacokinetic insights that might be crucial for the clinical utilization of BGM0504.

### Ethics declarations

Mice were euthanized using isoflurane inhalation followed by abdominal aorta exsanguinations. Rats were euthanized by inhalation of carbon dioxide. The cynomolgus monkeys were released from the study and were transferred to the stock/facility colony. Ethical approval PK animal experiments in this study have been approved by Institutional Animal Care and Use Committee of WuXi AppTec (Suzhou) Co., Ltd (approval no. SZ20210616-Rats-A, SZ20210519-Monkeys-B), Efficacy studies were approved by Institutional Animal Care and Use Committee of KCI Biotech Co., Ltd (approval no. 20210426-01, 20,210,511-01). All methods were performed in accordance with relevant regulations and guidelines including the ARRIVE guideline.

## Conclusion

In this work, we initially conducted a 500 ns molecular dynamics simulation study to examine the binding interactions of the non-acylated Tirzepatide with the GLP-1R/GIPR complex. Our findings revealed that in GLP-1R, E128ECD-K20 non-acylated Tirzepatide, and E127ECD-K20 non-acylated Tirzepatide can each form stable salt bridge. In GIPR, E119ECD-K20 non-acylated Tirzepatide was also observed to form a salt bridge. Identifying these electrostatic interactions elucidates the marked reduction in agonistic activity following the introduction of fatty acid moiety into the parent peptide. This observation implies that the modification strategy applied to Tirzepatide does not fully realize the agonistic potential inherent in the parent peptide.

Based on the outcomes of molecular dynamics simulations, we ascertained the feasibility of appending a K residue to the C-terminal of the parent peptide and subsequently introducing a new fatty acid moiety. Consequently, we devised a novel peptide agonist designated as BGM0504. Subsequent in vitro assays and in vivo experiments focused on the treatment of type II diabetes and the alleviation of NASH, and they demonstrated that BGM0504 exhibited superior efficacy compared to Tirzepatide. Notably, in vivo experiments, BGM0504 induced an acute reduction in body weight and food intake, which remained relatively stable throughout the later administration stages. The results suggest the potential therapeutic efficacy of BGM0504 in obesity indications, though such implications fall beyond the scope of this study.

To investigate the in vivo half-life of BGM0504, we conducted pharmacokinetic studies in SD rats and cynomolgus monkeys through intravenous administration and subcutaneous injection. The results of these studies indicated a prolonged half-life and elevated plasma exposure for BGM0504. These findings suggest that BGM0504 holds promise as a long-acting GLP-1R/GIPR dual agonist drug, providing valuable insights for potential application in subsequent clinical trials.

### Supplementary Information

Supplementary Information.

## Data Availability

Data is provided within the manuscript or supplementary information files; further inquiries can be directed to the author, Zheng Zheng: johnzhengzz@whut.edu.cn.
