# Supplementary material for: Molecular dynamics-guided optimization of BGM0504 enhances dual-target agonism for combating diabetes and obesity
Source: Sci Rep. 2024 Jul 19;14:16680. doi: 10.1038/s41598-024-66998-8 (PMC11271627; doi:10.1038/s41598-024-66998-8)
Supplement: Supplementary file 1 — Supplementary Information. [file 41598_2024_66998_MOESM1_ESM.docx]

**1. Molecular Dynamics Simulations Results**

1.1 Non-acylated Tirzepatide Binding to GLP-1R/GIPR


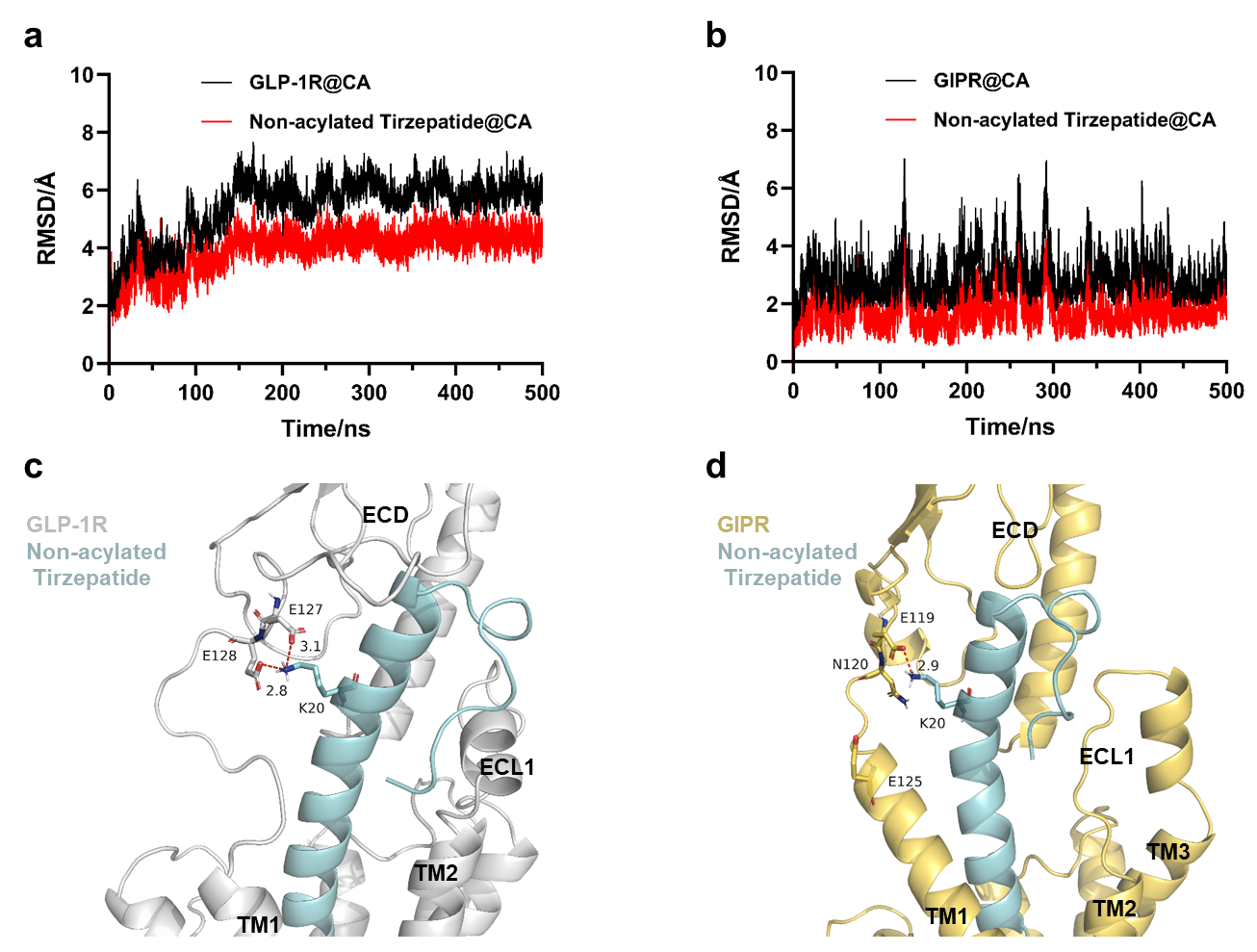


**Supplementary Figure 1.** (a) RMSD protein and peptide CA plot in the GLP-1R/Non-acylated Tirzepatide complex simulation. (b) RMSD plot of receptor and peptide CA in the GIPR/Non-acylated Tirzepatide complex simulation. (c) Conformation of GLP-1R/Non-acylated Tirzepatide complex simulation at 500 ns. (d) Conformation of GIPR/Non-acylated Tirzepatide complex simulation at 500 ns.

1.2 Tirzepatide and BGM0504 Binding to HSA protein


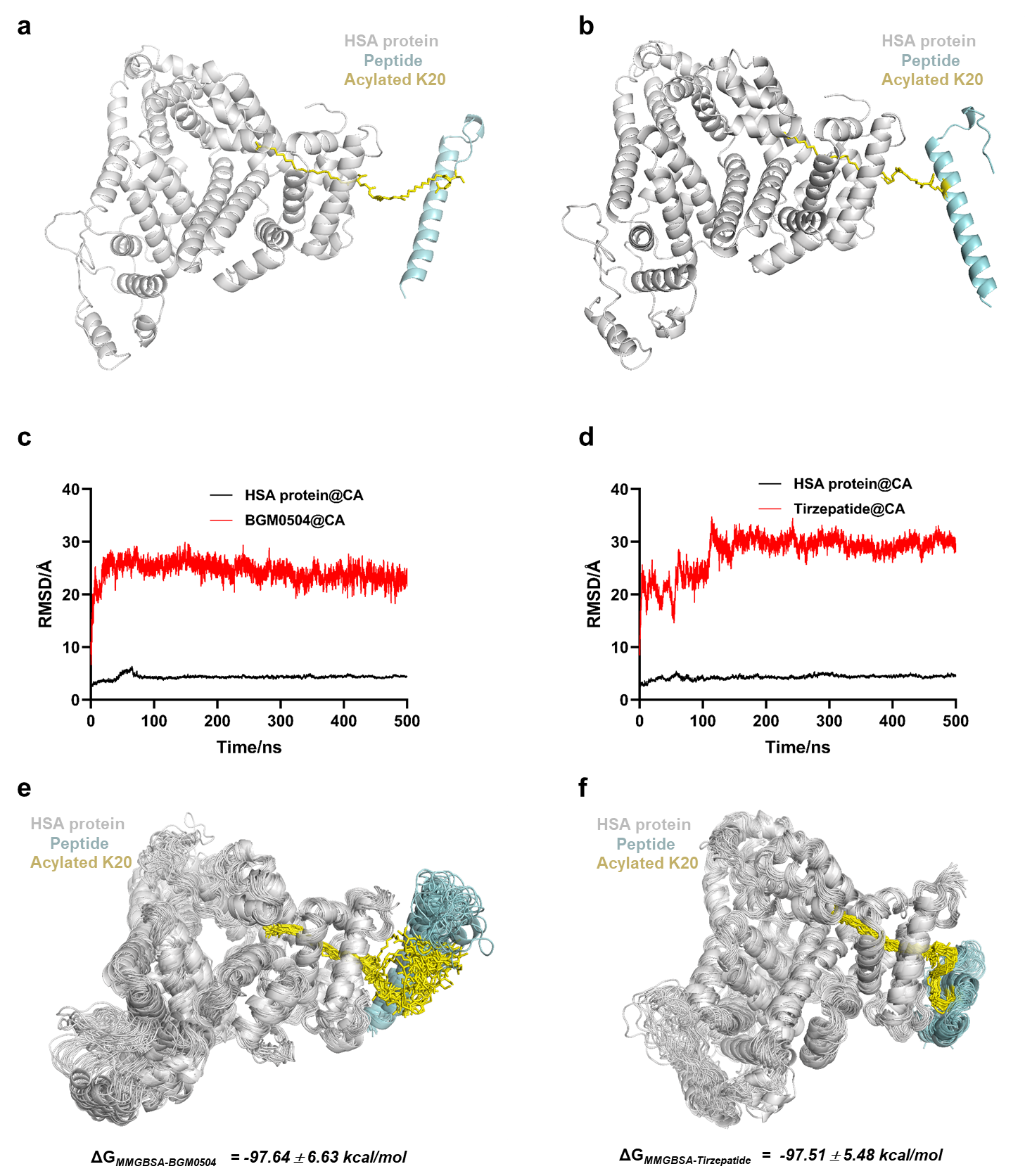


**Supplementary Figure 2.** (a) Structure of the Human Serum Albumin (HSA) protein/BGM0504 complex. (b) Structure of the HSA protein/Tirzepatide complex. (c) RMSD protein and peptide CA plot in the HSA protein/BGM0504 complex simulation. (d) RMSD plot of receptor and peptide CA plot in the HSA protein/Tirzepatide complex simulation. (e) Overlays of MD snapshots in BGM0504. Twenty snapshots of a complex system are displayed at 10 ns intervals from every 300~500 ns. (f) Overlays of molecular dynamics snapshots in Tirzepatide. Seven snapshots of a complex system are displayed at 10 ns intervals from every 300~500 ns.


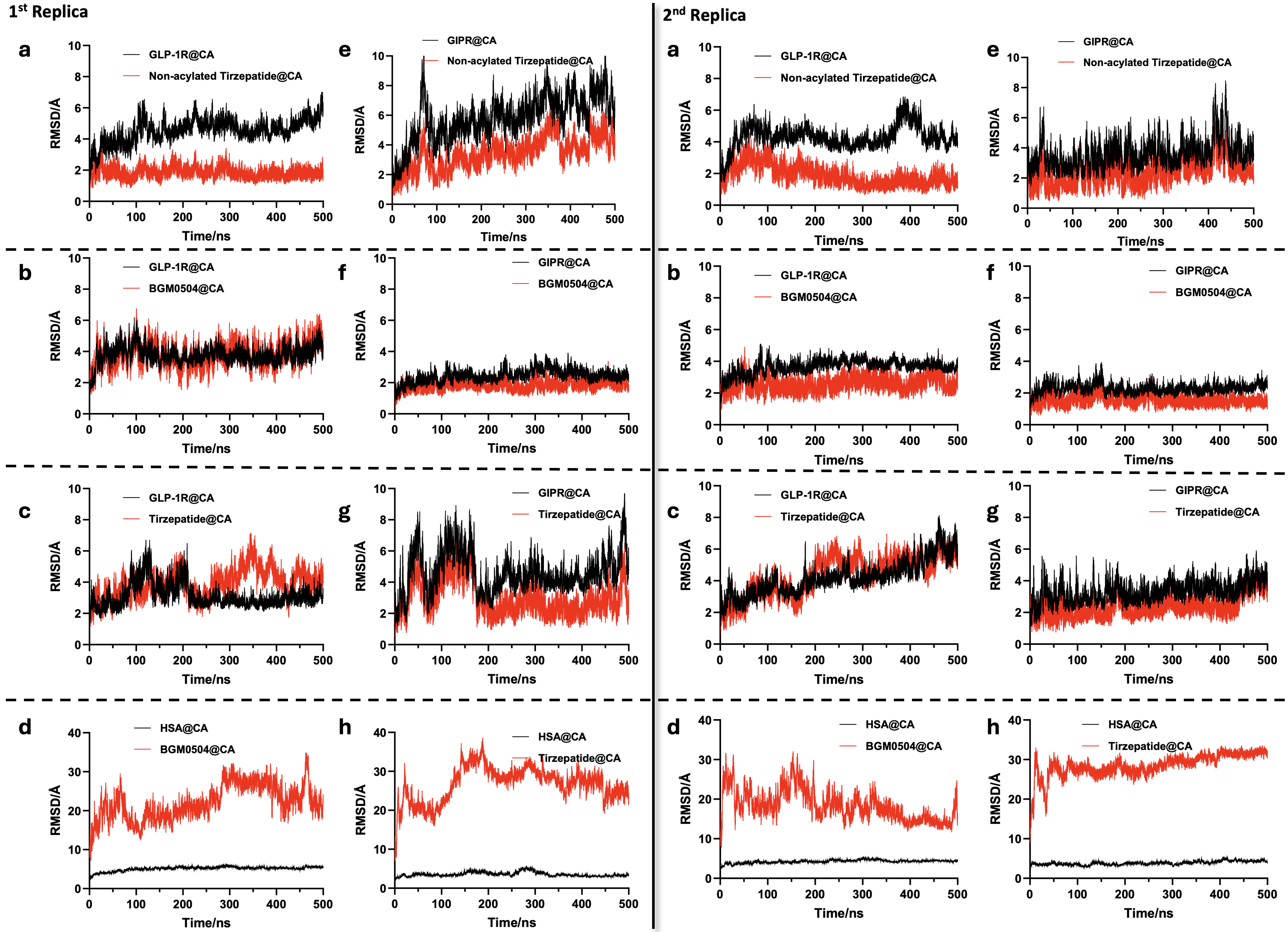


**Supplementary Figure 3.** Two more replica of the 500 ns MD simulations for the Non-acylated Tirzepatide, Tirzepatide and BGM0504 Binding to GLP-1R/GIPR proteins. a) RMSD protein and peptide CA plot in the GLP-1R/Non-acylated Tirzepatide complex simulation. b) RMSD protein and peptide CA plot in the GLP-1R/BGM0504 complex simulation. c) RMSD protein and peptide CA plot in the GLP-1R/Tirzepatide complex simulation. d) RMSD protein and peptide CA plot in the HSA/BGM0504 complex simulation. e) RMSD protein and peptide CA plot in the GIPR/Non-acylated Tirzepatide complex simulation. f) RMSD protein and peptide CA plot in the GIPR/BGM0504 complex simulation. g) RMSD protein and peptide CA plot in the GIPR/Tirzepatide complex simulation. h) RMSD protein and peptide CA plot in the HSA/Tirzepatide complex simulation.

**Supplementary Table 1.** Binding free energy and K20 salt bridges data (Mean±SD) averaged over three parallel MD simulations

| Sample ID | Human GLP-1R | | Human GIPR | |
| --- | --- | --- | --- | --- |
|  | ΔG_MMGBSA_ (kcal/mol) | K20 salt bridges occurrences, % | ΔGMMGBSA (kcal/mol) | K20 salt bridges occurrences, % |
| Tirzepatide | -161.38±18.47 | NA | -152.82±11.28 | NA |
| Non-acylated  Tirzepatide | -179.06±6.54 | 88.3±12.6  (to E128)  80.7±10.9  (to E127) | -160.59±5.29 | 66.5±8.8  (to E119) |
| BGM0504 | -182.39±11.72 | 93.1±7.9  (to E128)  92.5±3.92  (to E127) | -166.96±4.45 | 69.1±9.5  (to E119) |

ΔG_MMGBSA_ and K20 salt bridges occurrences value was indicated as geometric mean +/- SEM. NA: not available.

**2. In Vitro Experimental Evaluation**

**Supplementary Table 2.** cAMP accumulation assay data (Mean±SD)

| Sample ID | Human GLP-1R | | Human GIPR | |
| --- | --- | --- | --- | --- |
|  | EC_50_, nM±SEM (n) | E_max_, % | EC_50_, nM±SEM (n) | E_max_, % |
| Tirzepatide | 0.086±0.014 (3) | 84.1±5.0 | 0.441±0.141 (3) | 87.1±7.6 |
| Non-acylated  Tirzepatide | 0.031±0.005 (3) | 96.1±10.8 | 0.081±0.001 (3) | 92.2±1.7 |
| BGM0504 | 0.031±0.006 (3) | 92.7±7.6 | 0.182±0.011 (3) | 106.2±7.9 |
| GLP-1 (7-37) | 0.009±0.001 (3) | 102.2±6.7 | NA | NA |
| GIP | NA | NA | 0.177±0.046 (3) | 92.2±2.9 |

EC_50_ value was indicated as geometric mean +/- SEM, and n was the number of replicate assays. E_max_ was indicated as arithmetic mean +/- SEM. ND: not detected. NA: not available.


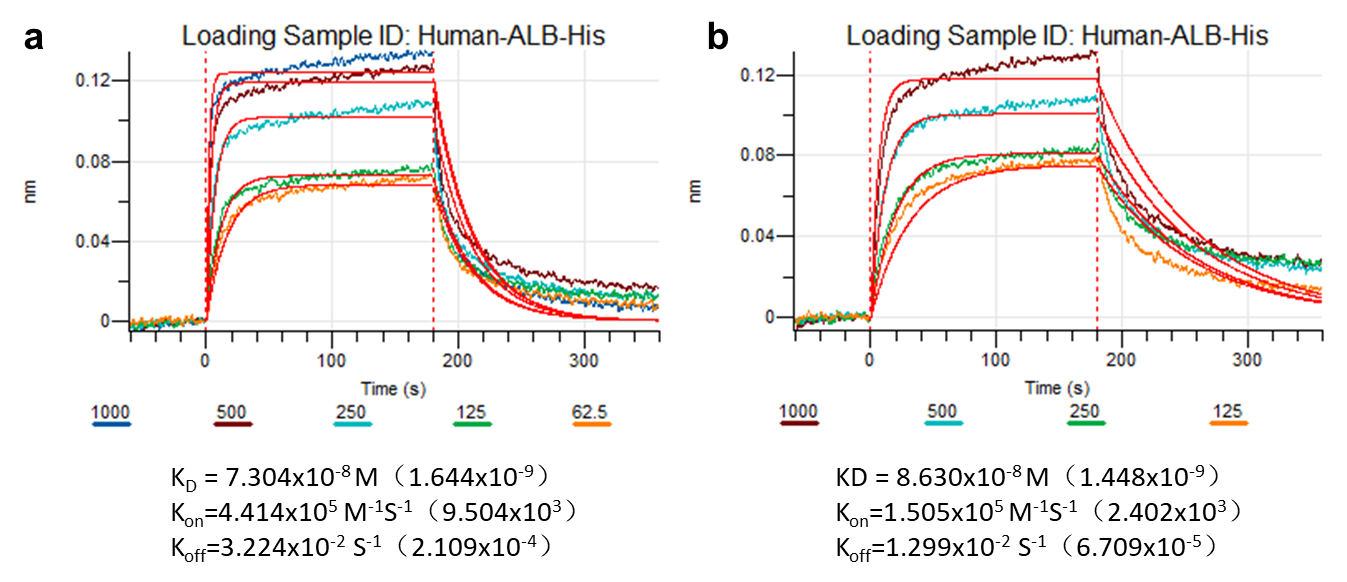


**Supplementary Figure 4.** (a) A BioLayer Interferometry (BLI) assay characterizing the binding between BGM0504 and Human-ALB-His. Color lines, model fits of BLI data from different concentrations of BGM0504. (b) A BLI assay characterizing the binding between Tirzepatide and Human-ALB-His. Color lines, model fits of BLI data from different concentrations of Tirzepatide.

**3. Evaluation Experiments of db/db Mice**

In the experiment involving db/db mice, the positive control group received a dosage of 0.15 mg/kg Tirzepatide, while the blank control group was subjected solely to a subcutaneous injection of the drug vehicle. To thoroughly investigate the dose dependence of BGM0504 in db/db mice, we established additional dose groups, including a low-dose group at 0.05 mg/kg and a high-dose group at 0.5 mg/kg. Each experimental group comprised 11 db/db mice. Following the initial administration, subsequent doses were administered every 3 days, spanning a total of 31 days. Throughout this study, we meticulously observed and analyzed a spectrum of parameters encompassing changes in body weight, food intake, non-fasting blood glucose post-initial administration.

The results delineating changes in animal body weight and the growth rate of body weight (Supplementary Figure 4A and B) revealed a pronounced downward trend in the weights of animals within each administration group during the initial treatment. Significantly, the degree of weight loss in the diverse BGM0504 administration groups exhibited a positive correlation with the dosage administered. Post day 3, the weight of animals in each treatment group resumed steady growth, with no discernible differences in body weight changes during the later stages of administration (p > 0.05). Supplementary Figure 4 visually conveys that the weight loss rate associated with BGM0504 surpasses that of Tirzepatide at equivalent doses. This underscores the superior efficacy of BGM0504 compared to the positive Tirzepatide control.


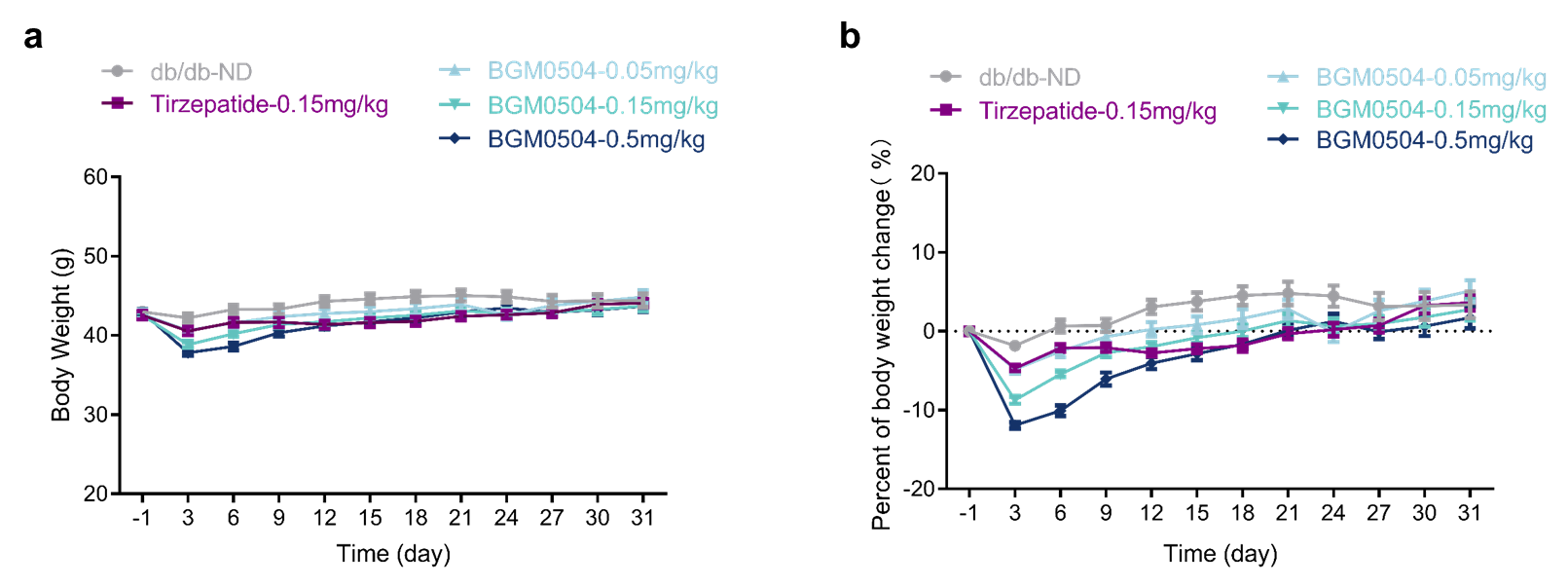


**Supplementary Figure 5.** (A) Body weight change was measured every 3 days. (B) The percent of body weight change was measured every 3 days.

The outcomes of alterations in animal food intake (Supplementary Figure 5) reveal a noteworthy decline when compared to the db/db-ND group, particularly evident after the initial dose across all treatment groups. The reduction in food intake within the various BGM0504 treatment groups exhibited a dose-dependent positive correlation. Post day 4, the food intake of animals in each drug group exhibited a resurgence, fluctuating within a narrow range and achieving a relatively stable state. Supplementary Figure 5 distinctly illustrates that the reduction in food intake within the BGM0504 administration group surpassed that observed in the positive control group (Tirzepatide) at equivalent doses, signifying a significantly more pronounced effect.

**
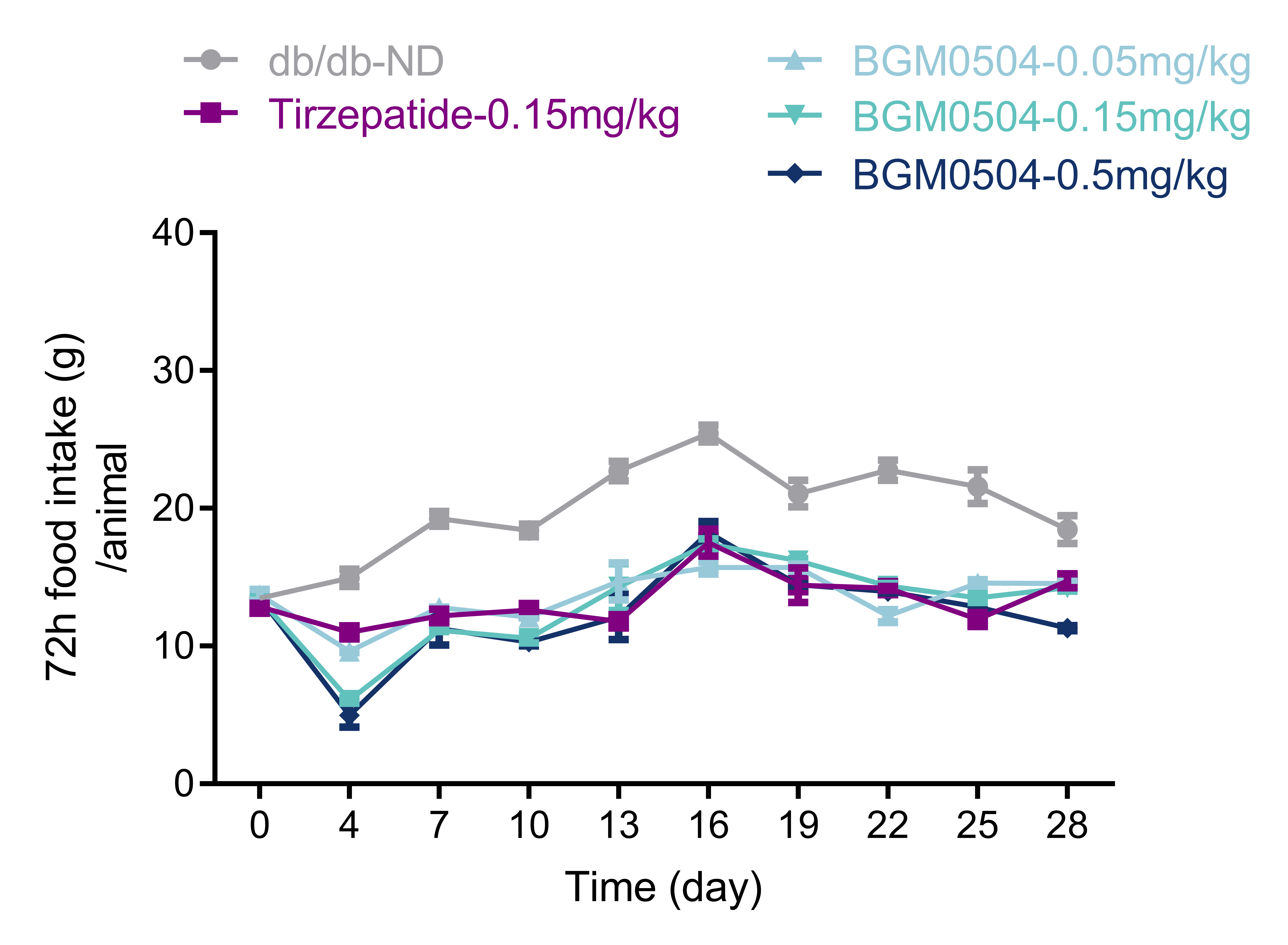
**

**Supplementary Figure 6.** Changes in food intake

**Supplementary Table 3.** Changes of insulin in serum (nIU/mL) (Mean±SD)

| Dose | db/db-ND  (n=11) | Tirzepatide-0.15mg/kg  (n=11) | BGM0504-0.05mg/kg  (n=11) | BGM0504-0.15mg/kg  (n=11) | BGM0504-0.5mg/kg  (n=11) |
| --- | --- | --- | --- | --- | --- |
| Insulin (nIU/mL) | 48991.0±3768.0 | 39227.0±2788.0 | 37649.0±3597.0^a^ | 30897.0±5634.0^a^ | 32774.0±6290.0^a^ |

*a: ^*^p<0.05 vs. db/db-ND, by T-test*

**4. Evaluation Experiment on C57 BL/6 Mice Induced by STZ+HFD**

4.1 Animal Weight

The results illustrating changes in animal weights (Supplementary Figure 6) demonstrate that following the initial administration, the weight of animals within each dosage group exhibited a marked decline. The extent of weight loss in various BGM0504 dosage groups displayed a positive correlation with the dosage. Post day 12, a notable downward trend in the weight of each dosage group persisted. While the weight of animals in these groups eventually resumed steady growth, it remained significantly lower than that of the model group. During the later stages of drug administration, no substantial differences in body weight changes among animals in each drug group were observed (p > 0.05).


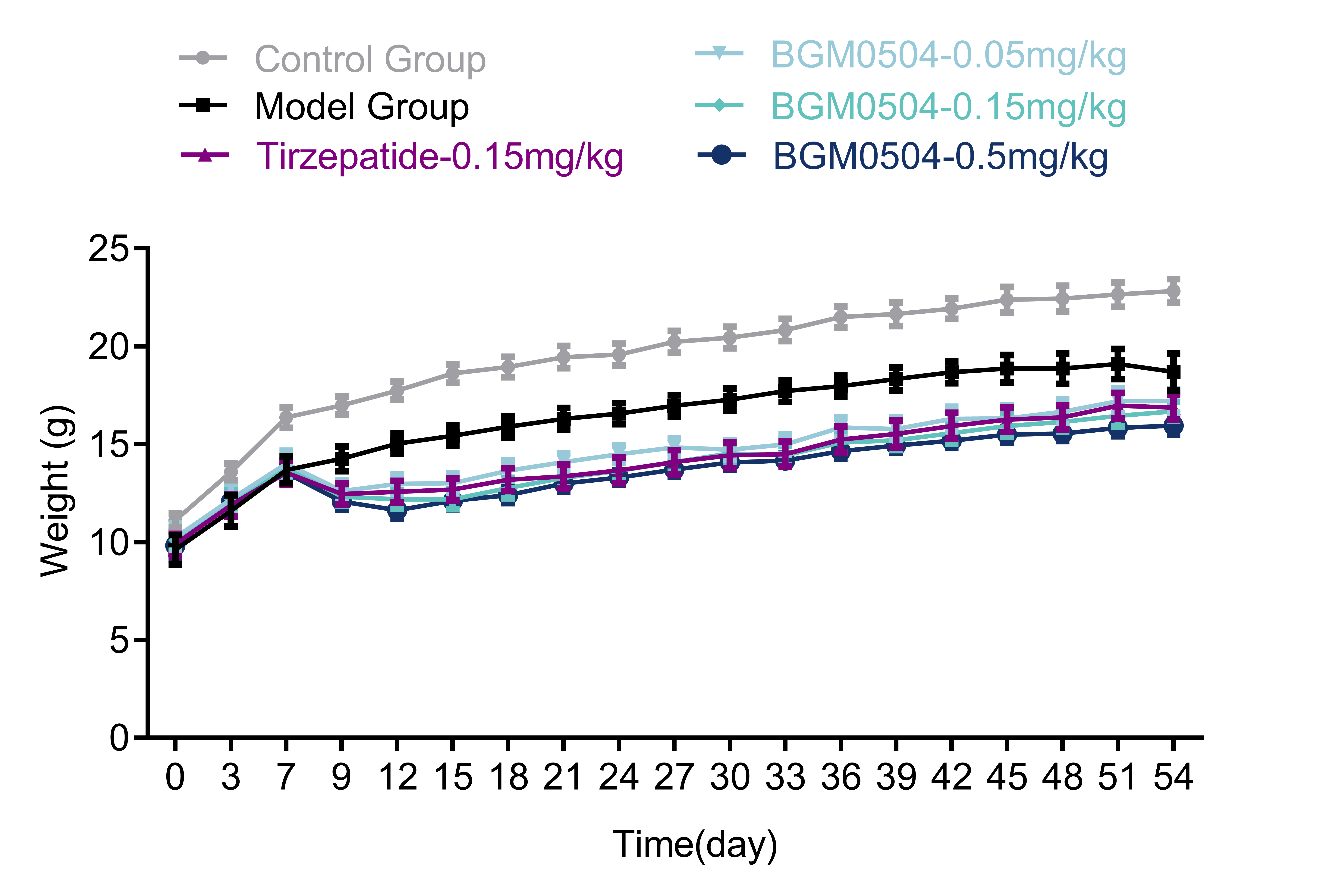


**Supplementary Figure 7.** Changes in animal weight

4.2 Food Intake

In comparison to the control group, the findings concerning alterations in animal food intake (Supplementary Figure 7) indicate that animals in each group exhibited lower food intake when provided with a high-fat diet (HFD) than animals in the control group given a normal diet. Throughout the entire testing period, no statistically significant differences in food intake were observed between the model group and each medication group (p > 0.05). Supplementary Figure 7 illustrates that the food intake of animals treated with BGM0504 is notably lower than that of animals treated with Tirzepatide at same dose group.


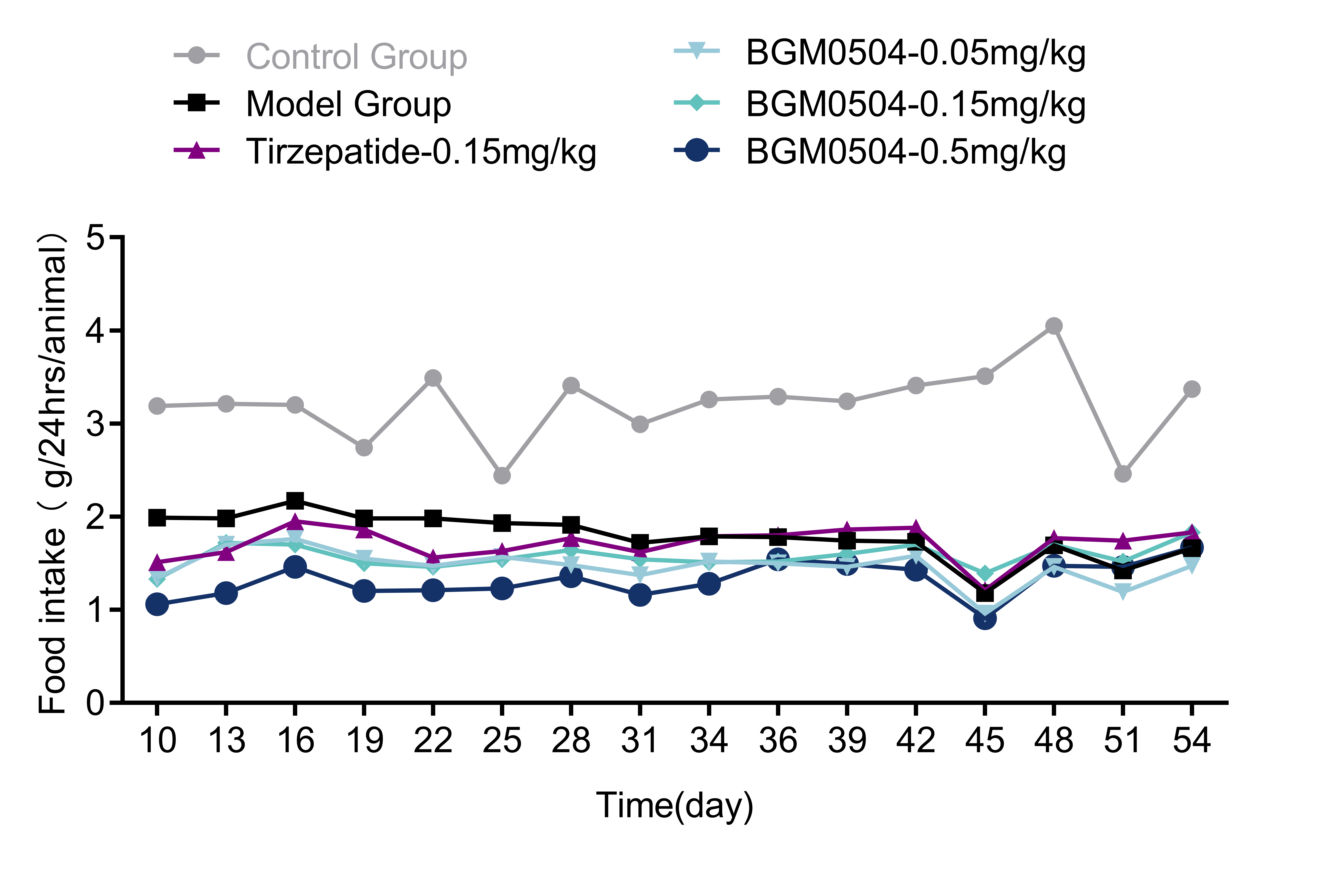


**Supplementary Figure 8.** Changes in food intake

4.3 Insulin

The serum insulin concentration in animals from the Tirzepatide-0.15 mg/kg group and each dose of the BGM0504 groups exhibited a decrease, with statistically significant reductions observed in the BGM0504-0.15 mg/kg and BGM0504-0.5 mg/kg groups compared to the db/db-ND group (p<0.05). Notably, the efficacy of BGM0504 within the same dosage group surpassed that of Tirzepatide.


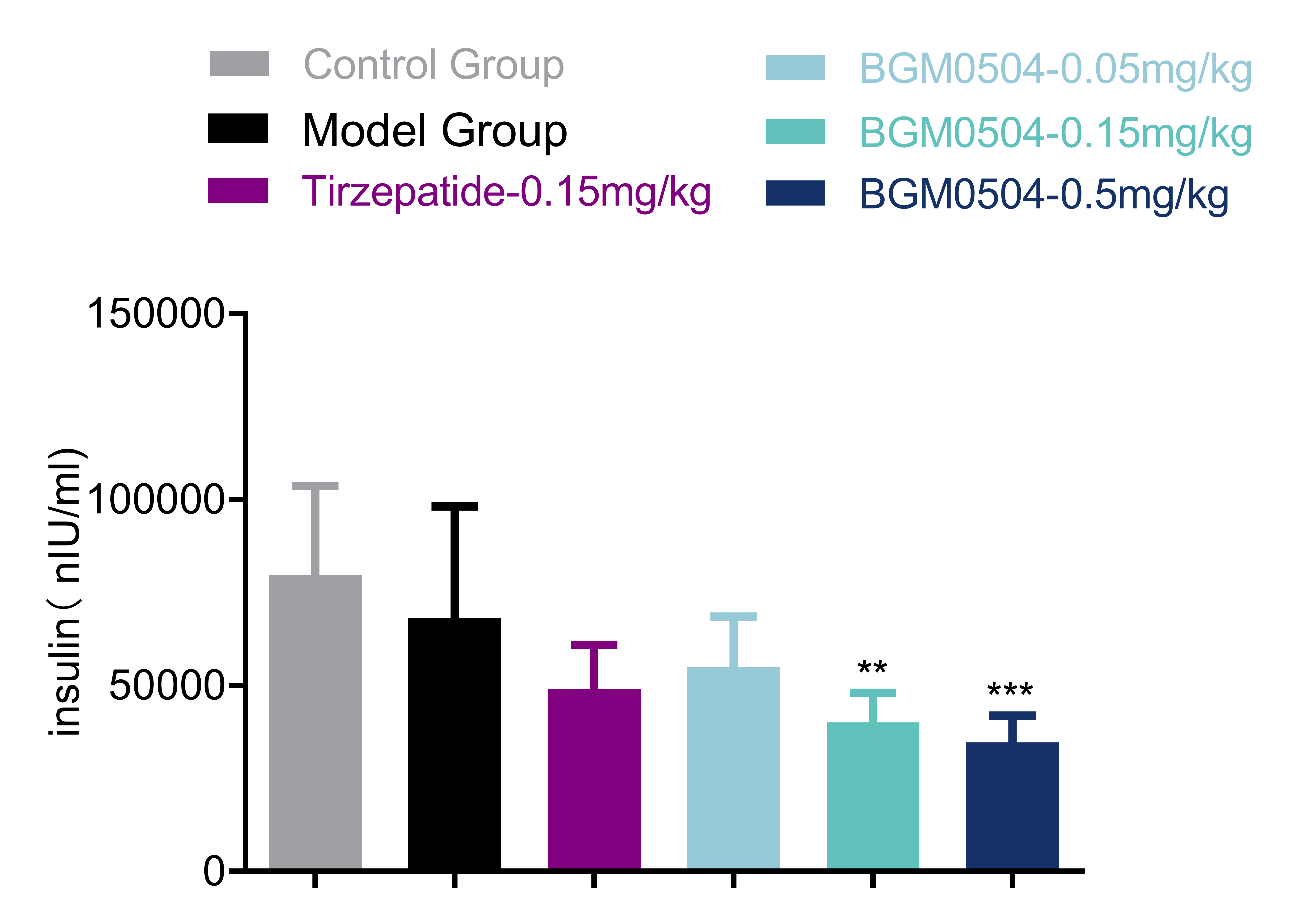


**Supplementary Figure 9.** Changes in insulin.

(One-Way ANOVA: **p<0.05 vs. model group；***p<0.001 vs. model group)

4.4 Biochemical Indicators Related to Liver Function and Blood Lipids

The results of liver function and blood lipid-related biochemical indicators are presented in Supplementary Table 2. Compared to the db/db-ND group, alanine transaminase (ALT) and aspartate aminotransferase (AST) exhibited a reduction in the Tirzepatide-0.15 mg/kg and BGM0504 groups at each dosage, with a significant difference in the decrease observed between the BGM0504-0.15 mg/kg and 0.5 mg/kg dose groups. Total cholesterol (TC) and triglycerides (TG) were significantly reduced in the Tirzepatide-0.15 mg/kg group and the various doses of BGM0504 groups. Low-density lipoprotein (LDL) showed a significant decrease in the Tirzepatide-0.15 mg/kg group and various doses of BGM0504 groups, while there was no significant change in high-density lipoprotein (HDL). Notably, the efficacy of BGM0504 within the same dosage group was superior to that of Tirzepatide.

**Supplementary Table 4.** Clinical biochemical indicators (Mean±SEM)

| Dose | Control Group | Model Group | Tirzepatide  0.15 mg/kg | BGM0504  0.05 mg/kg | BGM0504  0.15 mg/kg | BGM0504  0.5 mg/kg |
| --- | --- | --- | --- | --- | --- | --- |
|  | n=10 | n=9 | n=13 | n=11 | n=13 | n=13 |
| ALT (U/L) | 62.08±4.56^a^ | 104.67±7.22 | 79.23±10.51 | 73.97±9.20 | 67.58±7.73^a^ | 58.46±5.38^b^ |
| AST (U/L) | 89.55±4.70^c^ | 185.44±14.85 | 158.77±11.64 | 155.44±10.85 | 152.48±6.56^a^ | 150.54±6.26^a^ |
| TC (mmol/L) | 2.68±0.16^c^ | 7.64±1.98 | 3.58±0.21^c^ | 3.81±0.15^c^ | 3.25±0.12^c^ | 3.53±0.09^c^ |
| TG (mmol/L) | 0.83±0.02^b^ | 5.33±2.39 | 0.86±0.08^b^ | 1.09±0.20^b^ | 0.84±0.12^b^ | 0.88±0.14^b^ |
| HDL-c (mmol/L) | 1.76±0.25^a^ | 3.59±0.45 | 2.04±0.19 | 2.30±0.09 | 1.83±0.22 | 2.13±0.13 |
| LDL-c (mmol/L) | 0.45±0.05^c^ | 0.97±0.25 | 0.73±0.09^c^ | 0.56±0.03^b^ | 0.65±0.10^c^ | 0.62±0.03^c^ |

*a： ^*^p<0.05 vs. model group, by One-way ANOVA, Tukey’s test*

*b：^**^p<0.01 vs. model group, by One-way ANOVA, Tukey’s test*

*c： ^***^p<0.001 vs. model group, by One-way ANOVA, Tukey’s test*

*d: ^&&^p<0.01 vs. model group；e: ^&&&^p<0.01 vs. model group by T-test*

4.5 Liver Histopathology

4.5.1 NAS

**Supplementary Table 5.** Histopathological parameters (Mean±SEM)

|  | n | Steatosis | Inflammatory Cell Infiltration | Ballooning Degeneration | NAS |
| --- | --- | --- | --- | --- | --- |
| Control Group | 10 | 0.00±0.00^a^ | 0.00±0.00^a^ | 0.00±0.00 | 0.00±0.00^a^ |
| Model Group | 9 | 1.44±0.26 | 1.63±0.13 | 0.00±0.00 | 3.07±0.19 |
| Tirzepatide-0.15 mg/kg | 13 | 0.44±0.14^a^ | 1.05±0.07^a^ | 0.00±0.00 | 1.49±0.19^a^ |
| BGM0504-0.05 mg/kg | 11 | 0.52±0.12^a^ | 0.91±0.08^a^ | 0.00±0.00 | 1.42±0.14^a^ |
| BGM0504-0.15 mg/kg | 13 | 0.46±0.13^a^ | 0.90±0.06^a^ | 0.00±0.00^a^ | 1.36±0.15^a^ |
| BGM0504-0.5 mg/kg | 13 | 0.33±0.08^a^ | 1.03±0.05^a^ | 0.00±0.00 | 1.36±0.10^a^ |

*a： ^***^p<0.001 vs. model group, by One-way ANOVA，Tukey’s test*

4.5.2 Evaluation of Liver Fibrosis

**Supplementary Table 6.** Fibrosis percentage (Mean±SEM)

| Dose | Control Group | Model Group | Tirzepatide  0.15 mg/kg | BGM0504  0.05 mg/kg | BGM0504  0.15 mg/kg | BGM0504  0.5 mg/kg |
| --- | --- | --- | --- | --- | --- | --- |
|  | n=10 | n=9 | n=13 | n=11 | n=13 | n=13 |
| Fibrosis (%) | 0.61±0.03^c^ | 1.32±0.06 | 1.32±0.11 | 1.11±0.05^a^ | 1.10±0.04^b^ | 1.07±0.05^c^ |

*a： ^*^p<0.05 vs. model group, by T-test*

*b：^**^p<0.01 vs. model group, by T-test*

*c： ^***^p<0.001 vs. model group, by T-test*

**5. Pharmacokinetic Parameters**

After intravenous bolus injection of 0.2 mg/kg and subcutaneous injection of 0.3, 1.5, and 7.5 mg/kg of BGM0504 in male and female BLIague-Dawley (SD) rats, the average plasma pharmacokinetic parameters for both male and female subjects are presented in the table below.

**Supplementary Table 7.** Pharmacokinetic parameters of different doses of BGM0504 in SD rats

| Group | 1 | | 2 | | 3 | | 4 | |
| --- | --- | --- | --- | --- | --- | --- | --- | --- |
| Route of Administration | intravenous bolus injection | | subcutaneous injection | | subcutaneous injection | | subcutaneous injection | |
| Dose (mg/kg) | 0.2 | | 0.3 | | 1.5 | | 7.5 | |
| Pharmacokinetic Parameters | Mean | SEM | Mean | SEM | Mean | SEM | Mean | SEM |
| C0 or Cmax (ng/mL) | 4650 | 411 | 814 | 111 | 3830 | 705 | 15100 | 1750 |
| Tmax (h) | -- | -- | 24.0 | 0.00 | 24.0 | 0.00 | 20.0 | 6.20 |
| T1/2 (h) | 13.6 | 2.26 | 14.5 | 1.40 | 13.4 | 1.46 | 14.7 | 2.43 |
| Vdss (L/kg) | 0.0648 | 0.00579 | -- | -- | -- | -- | -- | -- |
| Cl (mL/min/kg) | 0.0680 | 0.0103 | -- | -- | -- | -- | -- | -- |
| AUC0-24 (ng·h/mL) | 38800 | 5150 | 14100 | 2440 | 63900 | 11400 | 269000 | 43000 |
| AUC0-last (ng·h/mL) | 49800 | 7470 | 33200 | 6190 | 153000 | 27700 | 637000 | 88700 |
| AUC0-inf (ng·h/mL) | 50000 | 7520 | 33400 | 6260 | 154000 | 28200 | 643000 | 92000 |
| Bioavailabilitya | -- | -- | 44.5% | -- | 41.1% | -- | 34.3% | -- |

--： not available.

After the intravenous bolus injection of 0.1 mg/kg and subcutaneous injection of 0.2, 1, and 5 mg/kg of BGM0504 in male and female experimental cynomolgus monkeys, the average plasma pharmacokinetic parameters for both male and female subjects are presented in the table below.

**Supplementary Table 8.** Pharmacokinetic parameters of different doses of BGM0504 in cynomolgus monkeys

| Group | 1 | | 2 | | 3 | | 4 | |
| --- | --- | --- | --- | --- | --- | --- | --- | --- |
| Route of Administration | intravenous bolus injection | | subcutaneous injection | | subcutaneous injection | | subcutaneous injection | |
| Dose (mg/kg) | 0.1 | | 0.2 | | 1 | | 5 | |
| Pharmacokinetic Parameters | Mean | SEM | Mean | SEM | Mean | SEM | Mean | SEM |
| C0 or Cmax (ng/mL) | 2860 | 261 | 1440 | 109 | 5970 | 1090 | 33500 | 3060 |
| Tmax (h) | -- | -- | 16.0 | 6.20 | 22.0 | 14.0 | 20.0 | 6.20 |
| T1/2 (h) | 37.9 | 2.46 | 38.7 | 1.84 | 41.7 | 2.92 | 43.4 | 5.92 |
| Vdss (L/kg) | 0.0608 | 0.00498 | -- | -- | -- | -- | -- | -- |
| Cl (mL/min/kg) | 0.0207 | 0.00216 | -- | -- | -- | -- | -- | -- |
| AUC0-24 (ng·h/mL) | 34600 | 2470 | 26100 | 1740 | 109000 | 21900 | 603000 | 94200 |
| AUC0-last (ng·h/mL) | 80400 | 7880 | 113000 | 7940 | 488000 | 51200 | 2720000 | 246000 |
| AUC0-inf (ng·h/mL) | 81100 | 7840 | 113000 | 7700 | 491000 | 51900 | 2740000 | 261000 |
| Bioavailabilitya | -- | -- | 69.7% | -- | 60.5% | -- | 67.6% | -- |

--： not available.


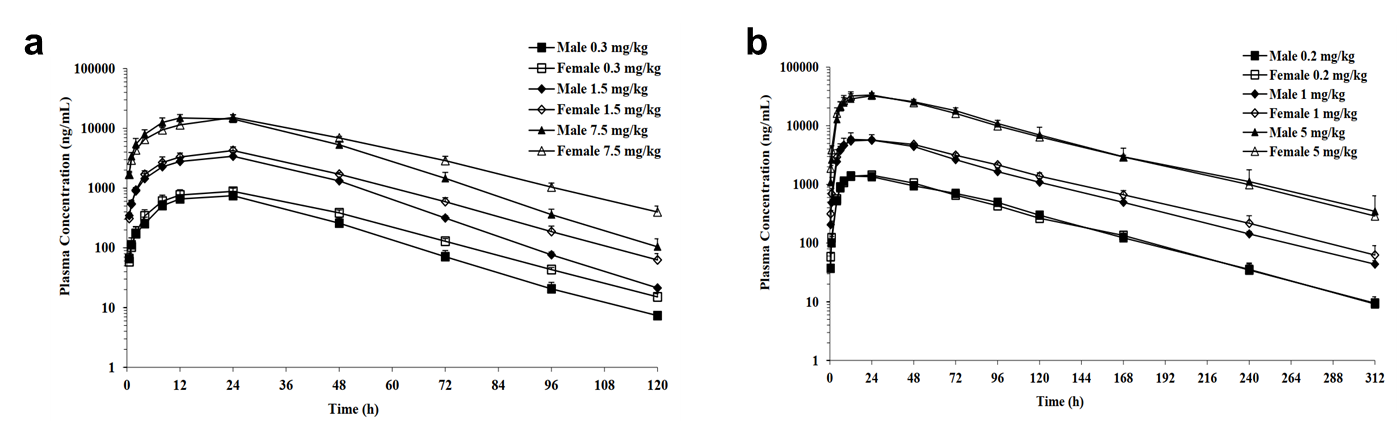


**Supplementary Figure 10.** (a) Mean plasma concentration profiles of BGM0504 in male and female SD rats following a single subcutaneous administration of BGM0504 at 0.3, 1.5, and 7.5 mg/kg. (b) Mean plasma concentration profiles of BGM0504 in male and female cynomolgus monkeys following a single subcutaneous administration of BGM0504 at 0.2, 1, and 5 mg/kg.

**6. Materials and Methods**

6.1 Molecular Dynamics Simulation

GLP-1R/GIPR complex system setup and simulation

Molecular dynamics (MD) simulations were performed using Amber18.^3^ The initial structures of the GLP-1R/non-acylated Tirzepatide complex and GIPR/non-acylated Tirzepatide complex were obtained from PDB codes 7VBI and 7FIY, respectively.^1^ The G protein and Nb35 were removed from the complexes using MOE2022.^2^ The N-termini of the peptides were capped with acetyl groups. The peptide-receptor complexes were embedded into a POPC lipid bilayer containing 195-200 lipids and solvated with explicit TIP3P water molecules containing 0.15 M NaCl using CHARMM-GUI Membrane Builder.^4^ The resulting PDB files were converted to Amber format using the charmmlipid2amber.py script. The protein, POPC membrane, and ligand molecules were parameterized using AMBERff15ipq-m,^5^ lipid14,^6^ and the general Amber force field 2 (GAFF2),^7^ respectively. The γGlu-2×OEG linker and C18 fatty diacid moiety attached to Lys20 in Tirzepatide, as well as the γGlu-3×PEG linker and C18 fatty diacid moiety attached to Lys40 in BGM0504, were modeled with the side chains extended into the solvent, keeping the initial pose of the side chains with no significant interactions with the solutes. Partial charges for these moieties were calculated using the Antechamber program with the AM1-BCC method.^8, 9^

In the MD simulations, all the covalent bonds containing hydrogen atoms were restrained using the SHAKE algorithm, and a time step of 2 fs was used.^10^ A cutoff of 10 Å was used for the non-bond interactions, and the PME method was used to handle the long-range electrostatic interaction.^11, 12^ The complex system and membrane was first relaxed using the steepest descent energy minimization, followed by slow heating to 310 K with restraints in the NVT ensemble. The complex system and membrane restraints were reduced gradually over 5 ns. Finally, a restrain-free production run was carried out for each simulation, with a time step of 2 fs in the NPT ensemble at 310 K. In this study, we conducted three parallel 500 ns simulations to ensure sufficient sampling. The CPPTRAJ module was used for the analysis of trajectories.^13^

HSA complex system setup and simulation

HSA protein/Tirzepatide complex and HSA protein/BGM0504 were modeled by PDB code (6YG9). In the initial complex conformation, the peptide portions of Tirzepatide and BGM0504 were completely solvated and do not interacted with HAS protein. The MOE2019 prepared the peptide-protein complexes. Molecular dynamics simulations were conducted using Amber18. The peptides were appropriately capped with acetyl groups. The complexes were solvated in explicit TIP3P waters. AMBERff15ipq-m and the general Amber force field 2 (GAFF2) were applied for the protein and ligand molecules. The partial charges of γGlu-2×OEG linker and C18 fatty diacid moiety and γGlu-2×3PEG linker and C18 fatty diacid moiety residues were calculated using the Antechamber program with the AM1-BCC method.

In the MD simulations, all the covalent bonds containing hydrogen atoms were restrained using the SHAKE algorithm, and a time step of 2 fs was used. A cutoff of 10 Å was used for the nonbond interactions, and the particle mesh Ewald (PME) method was used to handle the long-range electrostatic interaction. The complex system was first relaxed using the steepest descent energy minimization, followed by slow heating to 310 K with restraints. The complex system restraints were reduced gradually over 5 ns. Finally, a restrain-free production run was carried out for each simulation, with a time step of 2 fs in the NPT ensemble at 310 K. In this study, we conducted three parallel 500 ns simulations to ensure sufficient sampling. The cpptraj module was used for the analysis of trajectories.

The MMGBSA calculations were executed utilizing the MMPBSA.py module, which is included in the AmberTools package (version 18)^14^. These calculations employed the modified Generalized Born (GB) model formulated by Onufriev et al.^15^, incorporating a salt concentration of 0.15M. The analysis was based on a sampling of stable trajectories, taken at intervals of every 1 ns while excluding the final 100 ns from each simulation to ensure the reliability of the data.

6.2 Synthesis of Peptides

The solid-phase synthesis on Fmoc-Rink MBHA Amide resin followed a meticulously orchestrated sequence. The Fmoc protecting group was removed with a 20% piperidine/DMF solution, followed by the sequential coupling of the following Fmoc-protected amino acids: Fmoc-L-Lys(Alloc)-OH , Fmoc-L-Ser(tBu)-OH, Fmoc-L-Pro-OH, Fmoc-L-Pro-OH, Fmoc-L-Pro-OH, Fmoc-L-Ala-OH, Fmoc-Gly-OH, Fmoc-L-Ser(tBu)-OH, Fmoc-L-Ser(tBu)-OH, Fmoc-L-Pro-OH, Fmoc-Gly-OH, Fmoc-Gly-OH, Fmoc-L-Ala-OH, Fmoc-L-Ile-OH, Fmoc-L-Leu-OH, Fmoc-L-Trp(Boc)-OH, Fmoc-L-Gln(Trt)-OH, Fmoc-L-Val-OH, Fmoc-L-Phe-OH, Fmoc-L-Ala-OH, Fmoc-L-Lys(Boc)-OH, Fmoc-L-Gln(Trt)-OH, Fmoc-L-Ala-OH, Fmoc-L-Ile-OH, Fmoc-L-Lys(Boc)-OH, Fmoc-L-Asp(OtBu)-OH, Fmoc-L-Leu-OH, Fmoc-Aib-OH, Fmoc-L-Ile-OH, Fmoc-L-Ser(tBu)-OH, Fmoc-L-Tyr(tBu)-OH, Fmoc-L-Asp(OtBu)-OH, Fmoc-L-Ser(tBu)-OH, Fmoc-L-Thr(tBu)-OH, Fmoc-L-Phe-OH, Fmoc-L-Thr(tBu)-OH, Fmoc-Gly-OH, Fmoc-L-Glu(OtBu)-OH, Fmoc-Aib-OH, Boc-L-Tyr(tBu)-OH. This sequence yielded the BGM0504 backbone peptide resin. The Alloc protecting group on the Lys40 side chain was removed using tetrakis(triphenylphosphine)palladium/phenylsilane/dichloromethane, followed by the sequential coupling of Fmoc-AEEEA, Fmoc-AEEEA, Fmoc-L-Glu(OtBu), and tBuO-Ara(OH). After completing the coupling reactions, the resin was dried to obtain the BGM0504 resin peptide. The peptide was cleaved from the resin using TFA/TIS/water, then precipitated, filtered, and dried to obtain crude BGM0504. The crude product was purified by preparative reverse-phase HPLC, desalted, concentrated, and lyophilized to yield the final BGM0504 product.

The related substances in the BGM0504 sample were analyzed using the HPLC area normalization method, revealing a total impurity content of 1.4%. For high-resolution mass spectrometry (HRMS), the test sample was separated using an ultra-high performance liquid chromatography (UHPLC) system. The mobile phase A consisted of 0.1% formic acid in water, while the mobile phase B was 0.1% formic acid in acetonitrile. Gradient separation was performed at a wavelength of 214 nm. The primary mass spectrum of the target peak was deconvoluted using UNIFI software (version 1.8.2, Waters), and the monoisotopic molecular weight of the product was calculated. HRMS determined the monoisotopic molecular weight ([M+H]+) of the product (batch number: BGM0504-21080501) to be 5027.6883 Da. This is consistent with the theoretical molecular weight of the product, which is 5026.672 Da (molecular formula: C235H368N50O71), showing a mass deviation of 3.2 ppm. These results confirm that the analytical data matches the molecular weight of the product.

The synthesis steps for Non-acylated Tirzepatide and Tirzepatide were similar to those of BGM0504.

6.3 In vitro Functional Assays

To analyze the binding affinity to the human GLP-1 and GIP receptors, the prepared GLP-1R or GIPR membrane was first added to the assay plate, with a volume of 50 μL per well. Subsequently, each sample underwent serial dilution, and 25 μL of each diluted sample was added to the assay plate to be mixed with the membrane. The final detection ranges for each sample were identified. For GLP-1R, BGM0504 and BGM0501 started at a concentration of 1000 nM, undergoing a 4-fold 10-point serial dilution; GLP-1(7–37) began at 500 nM, also undergoing a 4-fold 10-point serial dilution. The low control was set at 500 nM GLP-1(7–37), while the high control was the assay buffer. For GIPR, BGM0504 and BGM0501 started at 30000 nM, undergoing a 4-fold 10-point serial dilution; GIP started at 300 nM, also undergoing a 4-fold 10-point serial dilution. The low control was 300 nM GIP, while the high control was the assay buffer. Next, [125I]-GLP-1 was prepared to a 320 pM working solution using an assay buffer, and 25 μL was added to each well of the assay plate, resulting in a final concentration of 80 pM. Similarly, [125I]-GIP was prepared to a 160 pM working solution, with 25 μL added to each well, achieving a final concentration of 40 pM. The assay plate was then sealed and incubated at room temperature for 1 hour on a shaker. The GF/C plate was soaked in the soaking buffer for at least 0.5 hours. Post-incubation, a cell harvester collected the reaction solution onto the GF/C plate. The plate was washed six times with a wash buffer, then dried in an oven at 50°C for 1 hour. The bottom of the GF/C plate was sealed, followed by adding 50 μL/well of MicroscintO cocktail, and then the top of the plate was sealed. The plate was read on a Microbeta2. For data analysis, each data point’s percentage inhibition was calculated as 100% minus the (sample signal - low control signal) divided by (high control signal - common control signal) times 100%. The ‘log(antagonist) vs. response – variable slope’ mode of GraphPad Prism 5.0 was used to fit the dose-response curve and calculate the IC50. Each sample underwent three runs.

To analyze the effect of BGM0504 on the GLP-1 and GIP receptors’ mediated cAMP pathway, cells exhibiting high expression of GLP-1 (HEK293 cells) or GIP (CHO cells) receptors were initially thawed, resuspended in Hank’s Balanced Salt Solution (HBSS), and dimethyl sulfoxide (DMSO) was removed via centrifugation. These cells were then resuspended in 5 mL of the assay buffer, and their concentration was determined using a Vi-Cell Counter. Specifically, cells expressing high levels of GLP-1 receptors were diluted to a concentration of 1.0×10^5 cells/mL. In contrast, those with increased expression of GIP receptors were cut to 2.0×10^5 cells/mL in the assay buffer. Samples were subsequently added to the assay plate along with ECHO555. The initial testing concentration for each sample was set at 100 nM for GLP-1 receptors and 500 nM for GIP receptors, followed by a 4-fold 10-point serial dilution. In the assay system, the testing range for GLP-1(7–37) started at 10 nM and for GIP at 200 nM, both undergoing a similar 4-fold 10-point serial dilution. The high control for GLP-1R testing was set at 10 nM GLP-1(7–37), and the low control at 1% DMSO. For GIPR testing, the high control was 200 nM GIP, with the standard control also at 1% DMSO. A volume of 10 µL of the cell suspension was added to the assay plate, with a density of 1000 cells/well for GLP-1R testing and 2000 cells/well for GIPR testing. The assay plate was then incubated in a 23°C incubator for 30 minutes. A starting concentration of 800 nM was used to prepare the cAMP standard curve, followed by a 4-fold 10-point serial dilution. These serially diluted cAMP standards were added to the assay plate at a volume of 10 µL/well. Subsequently, 10 µL of the cAMP detection solution was added to the assay plate, and the plate was incubated at room temperature for 1 hour, shielded from direct light. The assay plate was then read using an Envision system, with the final readout being the ratio of light emitted at 665 nm to 615 nm. For data analysis, each data point’s cAMP level (nM) was calculated using the established cAMP standard curve. The percentage activity for each data point was determined using the formula: % Activity = (cAMP level of testing sample - average cAMP level of low control) / (intermediate cAMP level of high control - average cAMP level of common control) × 100%. Additionally, the ‘log(agonist) vs. response – variable slope’ model in GraphPad Prism 5.0 was used to fit the dose-response curve of each sample and calculate the EC50 value.

To analyze the binding affinity to the HSA, purified Human ALB (25–609)-His was immobilized onto a HIS1K sensor chip at a concentration of 10 µg/ml, with an immobilization duration of 300 seconds, achieving a surface density of 0.1 nM. The buffer used was PBST (PBS with 0.02% Tween 20). BGM0504 was prepared in a series of dilutions at concentrations of 1000, 500, 250, 125, 62.5, and 0 nM, while Tirzepatide was diluted to concentrations of 2000, 1000, 500, 250, 125, and 0 nM for affinity analysis. For BLI measurements, the protocol for the affinity detection included a 60-second equilibration phase, a 180-second association phase, and a 180-second dissociation phase, all conducted at a controlled temperature of 25°C.

6.4 Experimental Assessment of Blood Glucose Reducing Effect in db/db Mice Model

In this experimental endeavor, we engaged 55 male db/db mice of SPF grade, procured at 6–8 weeks, from Zhejiang Vital River Laboratory Animal Technology Co., Ltd. The animals were divided into groups based on body weight and fasting blood glucose levels. Tirzepatide and BGM0504 were dissolved in a pH 7.0, 20mM sodium citrate buffer solution. The groups included the db/db-ND group, the Tirzepatide-0.15 mg/kg group, the BGM0504-0.05 mg/kg group, the BGM0504-0.15 mg/kg group, and the BGM0504-0.5 mg/kg group, each comprising 11 mice. Tirzepatide and BGM0504 were administered every 3 days. Non-fasting blood glucose levels were measured at 0, 1, 2, 3, 6, 24, 48, and 72 hours post-administration. Three days after the last dose, the mice were weighed. Subsequently, a portion of non-anticoagulated whole blood was collected from the orbital venous plexus under anesthesia. Blood samples were left at room temperature for 30 minutes, then centrifuged at 5000 rpm for 5 minutes. The serum was separated and frozen at -80°C for serum insulin content testing. Serum insulin levels were measured using the enzyme-linked immunosorbent assay (ELISA) method.

6.5 Experimental Assessment in STZ+HFD-Induced C57BL/6 Mice

This experiment involved selecting 30 SPF grade C57BL/6 pregnant female mice provided by Shanghai Jihui Experimental Animal Breeding Co., Ltd. Newborn male mice underwent subcutaneous injection of STZ (streptozotocin) (100 μg each) within 48 hours after birth and were nursed by their mothers for 4 weeks. During this period, we measured the fasting blood glucose of the mice and selected 65 diabetic male mice with fasting blood glucose values greater than or equal to 12 mmol/L as experimental animals. These mice were fed a HFD for 8 weeks to establish the STZ + HFD-induced diabetes and Nonalcoholic Steatohepatitis (NASH) model in male C57BL/6 mice. Fasting blood sugar was detected after 1 week of HFD feeding in these NASH model mice. Based on fasting blood sugar and body weight, they were randomly divided into the model group, the Tirzepatide-0.15 mg/kg group, the BGM0504-0.05 mg/kg group, the BGM0504-0.15 mg/kg group, and the BGM0504-0.5 mg/kg group. The treatment groups received administration once every 3 days. Additionally, their mothers nursed 10 newborn male mice without STZ injection for 4 weeks and then fed a regular maintenance diet as a control group. After HFD feeding, body weights were measured, and the animals’ food intake was recorded every 3 days until the end of the experiment. Three days after the last dose, animal weights were measured. Following a 6-hour fasting period, all experimental animals were anesthetized with an intraperitoneal injection of sodium pentobarbital. Non-anticoagulated whole blood was collected through the orbital venous plexus, left at room temperature for 30 minutes, and centrifuged at 5000 rpm for 5 minutes. The serum was separated and stored at -80°C for biochemical testing. After thawing the frozen serum samples, serum ALT, AST, TG, TC, HDL-c and LDL-c levels in whole blood were measured using a Hitachi 7060 automatic biochemical detector. Insulin content was determined using an enzyme-linked immunoassay kit.

After the dissection procedure, the liver was perfused with pre-cooled saline and then obtained and photographed. Its weight was also measured. Subsequently, the liver was immersed in 10% formalin for fixation and divided into two parts. One part underwent pathological hematoxylin and eosin (H&E) staining. The H&E staining was conducted on paraffin sections of all animal livers following the KCI pathology staining standard operating procedure (SOP). These stained sections were panoramically scanned using a NanoZoomer Digital Pathology scanner. Different fields of view were selected for observation at various magnifications, and the liver lobes in the sections were scored. The other part was subjected to pathological Sirius red (SR) staining.

Similarly, SR staining was performed on paraffin sections of the liver following the KCI pathology staining SOP. The stained sections were also panoramically scanned using the NanoZoomer Digital Pathology scanner. Subsequently, VIS7.0 software was utilized to analyze the liver fibrosis area in the panoramically examined SR-stained sections, and the percentage of the liver fibrosis area with the total section area was calculated.

6.6 Pharmacokinetics

6.6.1 Rats

In this experiment, a total of 24 rats (12 of each sex) were provided by Beijing Vitong Lever Experimental Animal Technology Co., Ltd. Each animal was identified using a tail mark and had its health status confirmed by Suzhou WuXi AppTec’s veterinary team before use. The animal room was monitored for temperature and humidity, with data recorded daily to ensure that the relative humidity was maintained at 40%–70% and the temperature was between 20–26°C. The lighting adopted a 12-hour alternating light and dark mode. Regular growth feed was provided daily, and animals had ready access to water. Before administration, each animal was observed for health and appearance. Post-administration, the condition of the animals was observed and documented before and after each blood collection point. The ensemble of 24 SD rats was divided into four groups, with three specimens of each gender in each group. Animals in group 1 received a single intravenous bolus of 0.2 mg/kg BGM0504, and groups 2, 3, and 4 received a single subcutaneous injection of 0.3, 1.5, and 7.5 mg/kg BGM0504 in 20 mM citrate buffered solution (pH=7.0±0.2), respectively. Blood samples were collected for pharmacokinetic analysis at specific time points (0.5, 1, 2, 4, 8, 12, 24, 48, 72, 96, and 120 hours) before and after dosing. At the same time, blood glucose levels were measured before and at 1, 4, 8, 12, and 24 hours after dosing. The collected blood samples were transferred to labeled commercial sample tubes containing K2-EDTA, centrifuged, and plasma was retrieved; some were used for analysis, and the other part was reserved as a backup. Plasma was transferred to pre-cooled centrifuge tubes, snap-frozen in dry ice, and stored in an ultra-low temperature freezer at -60°C or lower until transported to the Drug Evaluation Department of WuXi AppTec Shanghai Drug Development Co., Ltd. for analysis.

6.6.2 Monkeys

In this trial, 24 cynomolgus monkeys (12 of each sex) were provided by Hainan Jingang Biotechnology Co., Ltd. Each animal was identified using a unique code on the chest and cage box, and its health status was confirmed by the veterinary team of WuXi AppTec Suzhou Drug Development Co., Ltd. before use. Temperature and humidity were monitored in the animal room, with data recorded daily to ensure that the relative humidity was maintained at 40%–70% and the temperature stayed between 18–26°C. The ventilation was set to 10–20 exchanges per hour, and a 12-hour alternating light-dark cycle was adopted. Regular growth feed was provided daily, and the animals had constant access to water. Additionally, the test animals were provided with fruit daily to meet their nutritional needs. The 24 cynomolgus monkeys were randomly divided into four groups, with three monkeys of each sex per group. Animals in group 1 received a single intravenous bolus of 0.1 mg/kg BGM0504, while groups 2, 3, and 4 received a single subcutaneous injection of 0.2, 1, and 5 mg/kg BGM0504 in 20 mM citrate buffer solution (pH=7.0±0.2), respectively. Blood samples for pharmacokinetic (PK) analysis were collected at specific time points (0.5, 1, 4, 6, 8, 12, 24, 48, 72, 96, 120, 168, 240, and 312 hours) before and after dosing. At the same time, blood glucose levels were measured at 0.5, 4, 24, 48, and 72 hours after dosing. The collected blood samples were transferred to labeled commercial tubes containing K2-EDTA (0.85–1.15 mg), gently inverted several times to ensure mixing, and immediately placed on wet ice. Within 60 minutes of blood collection, the samples were centrifuged for 10 minutes at 3200×g at 2°–8°C. Plasma was divided into two aliquots (one for analysis and one for later use), transferred to labeled polypropylene tubes, immediately snap-frozen vertically in dry ice, and stored in an ultra-low temperature freezer at -60°C or lower until transported to the Drug Evaluation Department of WuXi AppTec Shanghai Drug Development Co., Ltd. for LC-MS/MS analysis.

6.7 Ethics declarations

Mice were euthanized using isoflurane inhalation followed by abdominal aorta exsanguinations. Rats were euthanized by inhalation of carbon dioxide. The cynomolgus monkeys were released from the study and were transferred to the stock/facility colony. Ethical approval PK animal experiments in this study have been approved by Institutional Animal Care and Use Committee of WuXi AppTec (Suzhou) Co., Ltd (approval no. SZ20210616-Rats-A, SZ20210519-Monkeys-B) ,Efficacy studies were approved by Institutional Animal Care and Use Committee of KCI Biotech Co., Ltd (approval no. 20210426-01, 20210511-01). All methods were performed in accordance with relevant regulations and guidelines including the ARRIVE guideline.

**Reference**

1. Zhao F*, et al.* Structural insights into multiplexed pharmacological actions of tirzepatide and peptide 20 at the GIP, GLP-1 or glucagon receptors. *Nat Commun* **13**, 1057 (2022).

2. Inc CCG. Molecular operating environment (MOE).). Chemical Computing Group Inc. Montreal, QC, Canada (2016).

3. Case D*, et al.* AMBER 2018, University of California, San Francisco. *Ta [Google Scholar]*, (2018).

4. Wu EL*, et al.* CHARMM‐GUI membrane builder toward realistic biological membrane simulations.). Wiley Online Library (2014).

5. Debiec KT, Cerutti DS, Baker LR, Gronenborn AM, Case DA, Chong LT. Further along the road less traveled: AMBER ff15ipq, an original protein force field built on a self-consistent physical model. *Journal of chemical theory and computation* **12**, 3926-3947 (2016).

6. Dickson CJ*, et al.* Lipid14: The Amber Lipid Force Field. *J Chem Theory Comput* **10**, 865-879 (2014).

7. He X, Man VH, Yang W, Lee TS, Wang J. A fast and high-quality charge model for the next generation general AMBER force field. *J Chem Phys* **153**, 114502 (2020).

8. Jakalian A, Bush BL, Jack DB, Bayly CI. Fast, efficient generation of high‐quality atomic charges. AM1‐BCC model: I. Method. *Journal of computational chemistry* **21**, 132-146 (2000).

9. Jakalian A, Jack DB, Bayly CI. Fast, efficient generation of high-quality atomic charges. AM1-BCC model: II. Parameterization and validation. *J Comput Chem* **23**, 1623-1641 (2002).

10. Ryckaert J-P, Ciccotti G, Berendsen HJ. Numerical integration of the cartesian equations of motion of a system with constraints: molecular dynamics of n-alkanes. *Journal of computational physics* **23**, 327-341 (1977).

11. Darden T, York D, Pedersen L. Particle mesh Ewald: An N⋅ log (N) method for Ewald sums in large systems. *The Journal of chemical physics* **98**, 10089-10092 (1993).

12. Essmann U, Perera L, Berkowitz ML, Darden T, Lee H, Pedersen LG. A smooth particle mesh Ewald method. *The Journal of chemical physics* **103**, 8577-8593 (1995).

13. Roe DR, Cheatham TE, 3rd. PTRAJ and CPPTRAJ: Software for Processing and Analysis of Molecular Dynamics Trajectory Data. *J Chem Theory Comput* **9**, 3084-3095 (2013).

14. Miller BR, III, McGee TD, Jr., Swails JM, Homeyer N, Gohlke H, Roitberg AE. MMPBSA.py: An Efficient Program for End-State Free Energy Calculations. *Journal of Chemical Theory and Computation* **8**, 3314-3321 (2012).

15. Mongan J, Simmerling C, McCammon JA, Case DA, Onufriev A. Generalized Born model with a simple, robust molecular volume correction. *J Chem Theory Comput* **3**, 156-169 (2007).
